# A weld point cloud recognition method based on an improved Light Gradient Boosting Machine

**DOI:** 10.1038/s41598-026-54597-8

**Published:** 2026-05-28

**Authors:** Hongtao Yang, Ziqiang Bi, Xiulan Li, Qingan Yao, Peng Zhang, Haotian Bai, Yunlong Cheng

**Affiliations:** 1 School of Electrical and Electronic Engineering, Changchun University of Technology, Changchun, 130012 Jilin China; 2 College of Computer Science and Engineering, Changchun University of Technology, Changchun, 130012 Jilin China; 3 Engineering Technology Department, Tianjin CPC Keyuan Petroleum Engineering Co., Ltd, Tianjin, 300280 China

**Keywords:** 3D vision, Weld recognition, Point cloud processing, LightGBM, Metaheuristic optimization, Explainable artificial intelligence, Engineering, Mathematics and computing

## Abstract

Accurate weld-region identification is essential for weld quality inspection and automated grinding. However, weld point clouds are highly irregular and lack explicit topological structure, which makes accurate recognition challenging. To address this issue, this study formulates weld point-cloud recognition as a binary point-wise classification task. Each point is classified as either weld bead or base metal. A systematic classification framework is established by combining neighborhood-based geometric feature extraction, baseline model comparison, and metaheuristic hyperparameter optimization. Three morphology-specific weld subsets, including straight-line, curved-line, and S-shaped welds, are used for evaluation. The classification performance of Random Forest (RF), Extreme Gradient Boosting (XGBoost), and Light Gradient Boosting Machine (LightGBM) is first compared under different neighborhood scales. Overall Accuracy (OA), Precision, Recall, and F1-Score are used as evaluation metrics. The results show that LightGBM achieves the best baseline performance at a neighborhood radius of 1.5 mm. To further improve classification performance, LightGBM hyperparameters are optimized using metaheuristic algorithms. The compared optimizers include the Artificial Lemming Algorithm (ALA), Alpha Evolution Algorithm (AE), and Starfish Optimization Algorithm (SFOA). Repeated-run results demonstrate that AE-LightGBM achieves the most favorable overall performance under the unified evaluation protocol. Statistical significance analysis and convergence analysis further support the effectiveness of AE among the compared optimizers. In addition, SHapley Additive exPlanations (SHAP) is employed to analyze feature contributions and improve the interpretability of the optimized model. The proposed method provides an effective technical pathway for robot-based weld recognition and grinding tasks using 3D vision and supervised machine learning.

## Introduction

With the rapid development of the manufacturing industry, welding technology has been widely applied in numerous fields such as aerospace, automotive manufacturing, and ship processing^1–3^. Due to the irregularity and inaccuracy of weld seams after welding, further processing is often required to meet the standards of high-quality products. Robotic grinding is increasingly being utilized in grinding tasks due to its advantages of high flexibility, efficiency, and low cost^4,5^. Nevertheless, efficient and accurate weld-seam recognition from 3D point clouds remains challenging. It is therefore still an active research topic in robotic weld processing.

Vision sensors have been widely used in the field of robotic vision welding in recent years due to their advantages of being non-contact, high-precision, and high-speed processing^6,7^. Robotic vision measurement methods are mainly divided into two categories: active vision perception and passive vision perception. Passive vision sensors typically perform weld seam identification based on 2D images. For example, H.N.M. Shah et al. extracted the weld seam region by local threshold segmentation^8^, thereby identifying the weld seam area. Ang et al. employed the YOLOv3 model for end-to-end recognition and localization of weld beads in images^9^. Zou and Chen studied the feature recognition of lap joints through image processing and convolution operators^10^. However, these methods inherently rely on 2D pixel information and are sensitive to lighting conditions, surface reflection of workpieces, and camera perspective. Their robustness in complex industrial environments remains limited. As a result, they are difficult to adapt stably to the geometric variations of 3D weld seams. In contrast, active vision sensors project lasers or structured light onto the weld seam surface, fundamentally reducing dependence on ambient lighting. For instance, Ye et al. fitted the base material contour points obtained from a single-line laser sensor using an expectation-maximization algorithm to extract the weld bead contour^11^. Zhou et al. used the Random Sample Consensus (RANSAC) algorithm to analyze surface points collected by a laser vision sensor^12^. Weld bead width and height were then measured through multi-linear model fitting. However, the aforementioned traditional methods are mostly based on threshold segmentation, and their performance is significantly affected by threshold settings, often resulting in under-segmentation or over-segmentation.

With the development of machine learning, machine learning-based point cloud recognition methods have been extensively explored. Three-dimensional geometric features and machine learning algorithms have been applied to complex tasks, such as building roof segmentation from LiDAR point clouds^13^ or semantic labeling of mobile laser scanner point clouds^14^. Atik et al. employed a method combining multi-scale geometric features with machine learning for the classification of aerial point clouds^15^. Koray Aksu et al. extracted structural elements from indoor point clouds by comparing multiple machine learning models with the SHAP explainer^16^. Recent studies have further confirmed the importance of geometric information in 3D point-cloud classification and representation. Si and Wei investigated feature extraction from 3D point-cloud data and showed that local neighborhood encoding can improve the representation of point-cloud structures^17^. Other studies also reported that incorporating geometric attributes and multi-feature information can enhance the representational capacity and classification robustness of point-cloud models^18,19^. This provides support for the use of local geometric descriptors in weld point-cloud recognition, where the weld bead and base metal are mainly distinguished by local surface morphology, height variation, and boundary characteristics. However, research on applying machine learning and 3D geometric features to weld seam point cloud recognition remains relatively limited. Rodriguez-Gonzalvez et al. first introduced 3D geometric features and machine learning into weld seam identification^20^, but this method still suffers from sensitivity to ambient lighting and high computational cost for point cloud acquisition. Kewen Huang et al. utilized a RealSense depth camera combined with machine learning algorithms for weld seam segmentation^21^. Although this partially mitigated the limitations of traditional camera methods, the effectiveness of geometric features was not deeply explored. Traditional machine learning techniques provide important insights for point cloud classification^22^. Among them, ensemble learning, which combines multiple base learners to construct a strong learner, has demonstrated excellent performance in numerous tasks^23^. Among various ensemble learning methods, gradient boosting machines iteratively combine multiple weak classifiers to build a strong classifier. In each iteration, the model corrects the residuals of the previous learner, thereby continuously improving prediction performance. This progressive optimization mechanism makes gradient boosting machines significantly outperform traditional models like Random Forest^24^ in classification accuracy. Within the gradient boosting decision tree framework, XGBoost has been gradually applied to 3D point cloud classification due to its high accuracy^25^. As a later development, LightGBM significantly improves computational efficiency while maintaining high accuracy^26^. XGBoost and LightGBM have therefore been widely used in large-scale point-cloud classification tasks. In addition to prediction accuracy, recent studies have increasingly emphasized the combination of GBDT-based models with model optimization and interpretability analysis. Related works have shown that LightGBM can be combined with SHAP-based interpretation to improve both predictive analysis and feature-contribution explanation^27^. In addition, metaheuristic-optimized LightGBM has also been explored in recent data-driven prediction tasks, demonstrating the potential of combining LightGBM with intelligent optimization and SHAP-based explanation^28^. These studies provide useful support for constructing an accurate and interpretable LightGBM-based framework in the present weld point-cloud recognition task.

However, the performance of GBDT-based models is strongly affected by their hyperparameter settings. For LightGBM, parameters such as the number of boosting trees, learning rate, maximum tree depth, and number of leaves jointly determine model complexity, convergence behavior, and generalization ability. Traditional search strategies, such as grid search and random search, are often inefficient in continuous and coupled hyperparameter spaces. Therefore, metaheuristic optimization provides a feasible strategy for automatically searching suitable LightGBM hyperparameter combinations^29^.

Among the candidate metaheuristic optimizers, AE is selected as the main optimizer in this study. Its Alpha operator combines adaptive base-vector construction, random perturbation, and adaptive step-size adjustment. This search mechanism enables candidate solutions to be updated by using both population information and adaptive step control. It is therefore beneficial for balancing global exploration and local exploitation. Since the key hyperparameters of LightGBM are coupled and jointly affect classification performance, AE is expected to provide an effective search strategy for identifying suitable parameter combinations. To provide a comparative evaluation, ALA and SFOA are also included as reference optimizers under the same optimization protocol.

Therefore, this study adopts LightGBM for weld point-cloud classification, further introduces intelligent optimization algorithms for hyperparameter tuning, and employs the SHAP explainability framework to analyze the relative importance of input geometric features. In this way, the study aims to improve classification performance while enhancing the interpretability of the machine learning model. The main contributions of this study are summarized as follows:

1. Construction of a weld point-cloud dataset and baseline model evaluation.

A visual robotic experimental platform was built to acquire 3D point-cloud data of weld seams with three representative morphologies, namely straight-line, curved-line, and S-shaped welds. Geometric features were extracted under four neighborhood radii: 0.5 mm, 1.0 mm, 1.5 mm, and 2.0 mm. These features were used to construct a weld point-cloud dataset for the binary point-wise classification of weld bead versus base metal. On this basis, three machine learning models, including Random Forest (RF), XGBoost, and LightGBM, were systematically evaluated. The results show that LightGBM achieved the best baseline classification performance at a neighborhood radius of 1.5 mm.

2. Improvement of LightGBM through hyperparameter optimization.

To further improve classification performance, AE was introduced as the main optimizer for LightGBM hyperparameter tuning because of its adaptive search mechanism based on the Alpha operator. ALA and SFOA were also included as comparative optimizers. All optimizers were evaluated using the same search space, optimization budget, validation strategy, and repeated-run setting. The results show that AE-LightGBM achieved the best overall performance among the optimized models, and statistical significance analysis and convergence analysis further support the effectiveness of AE for the current weld point-cloud classification task.

3. Interpretability analysis of the model decision mechanism.

The SHAP explainability framework was employed to analyze the feature contributions of AE-LightGBM and to clarify the influence of different geometric features on the classification results. This analysis improves the interpretability of the model and provides additional support for its application in practical weld recognition and grinding tasks.

## Materials and methods

### Experimental setup

The weld seam point cloud acquisition system employed in this paper consists of an AUBO-E5 collaborative robot (including controller and teach pendant) and an S028120 area structured light camera. The camera has a resolution of 1280 pixels × 960 pixels and a single-point repeatability accuracy of 0.0012 mm, enabling it to directly obtain the 3D point cloud coordinates of the target. The system collected point cloud data for three types of weld beads—straight-line, curved-line, and S-shaped—by controlling the robotic arm through the teach pendant. The collected data were then transmitted via the control cabinet to the host computer for subsequent processing.

**Fig. 1:**
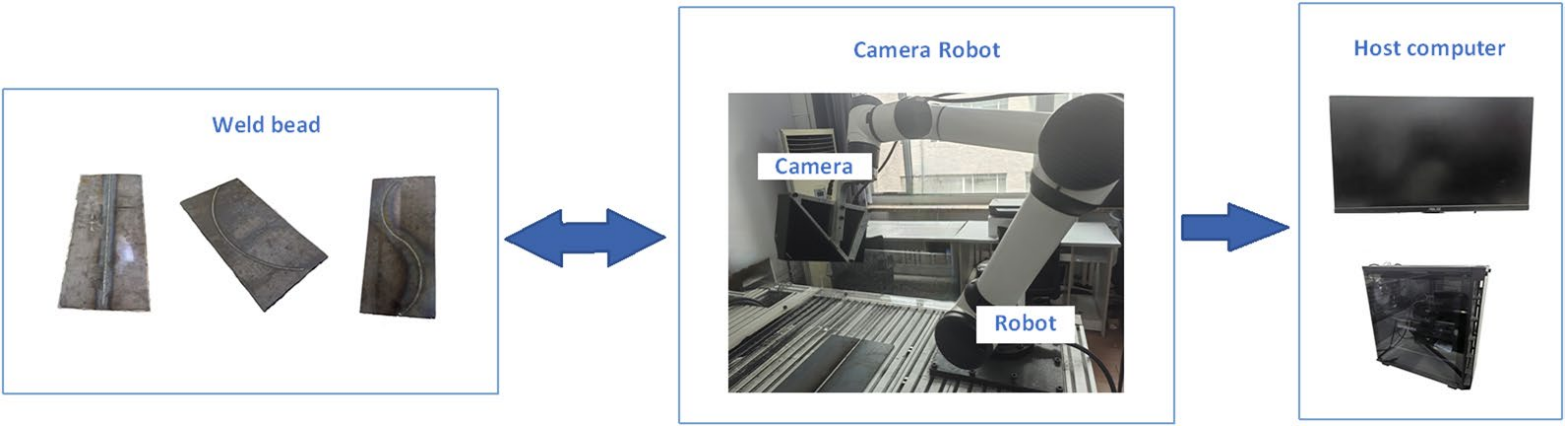
Weld-seam point-cloud acquisition system, including the robot, structured-light camera, welded plate, and host computer.

During data acquisition, all welded plates were placed on a stable workbench, and the structured-light camera was fixed relative to the workpiece during each scan. The camera was positioned approximately 200 mm above the base-metal surface. The experiments were conducted under stable indoor laboratory illumination. Direct sunlight and strong external light sources were avoided during acquisition, thereby reducing illumination variation, surface reflection, and potential interference with structured-light imaging. To reduce the influence of vibration, point-cloud acquisition was performed after the robot reached the target scanning pose and remained stationary. No additional mechanical vibration was intentionally introduced during data collection. These controlled acquisition conditions helped improve the repeatability of the acquired point clouds and reduced the influence of environmental disturbances on geometric feature extraction. The composition of the entire point-cloud acquisition system is illustrated in Fig. 1.

### Task definition and dataset overview

This study focuses on a binary point-wise classification task for weld point clouds, with the objective of assigning each point within the weld region to one of two categories: weld bead or base metal. As a typical point-wise classification problem in point cloud analysis, this task provides fundamental support for subsequent applications such as weld identification, quality evaluation, and automated grinding path planning.

**Fig. 2:**
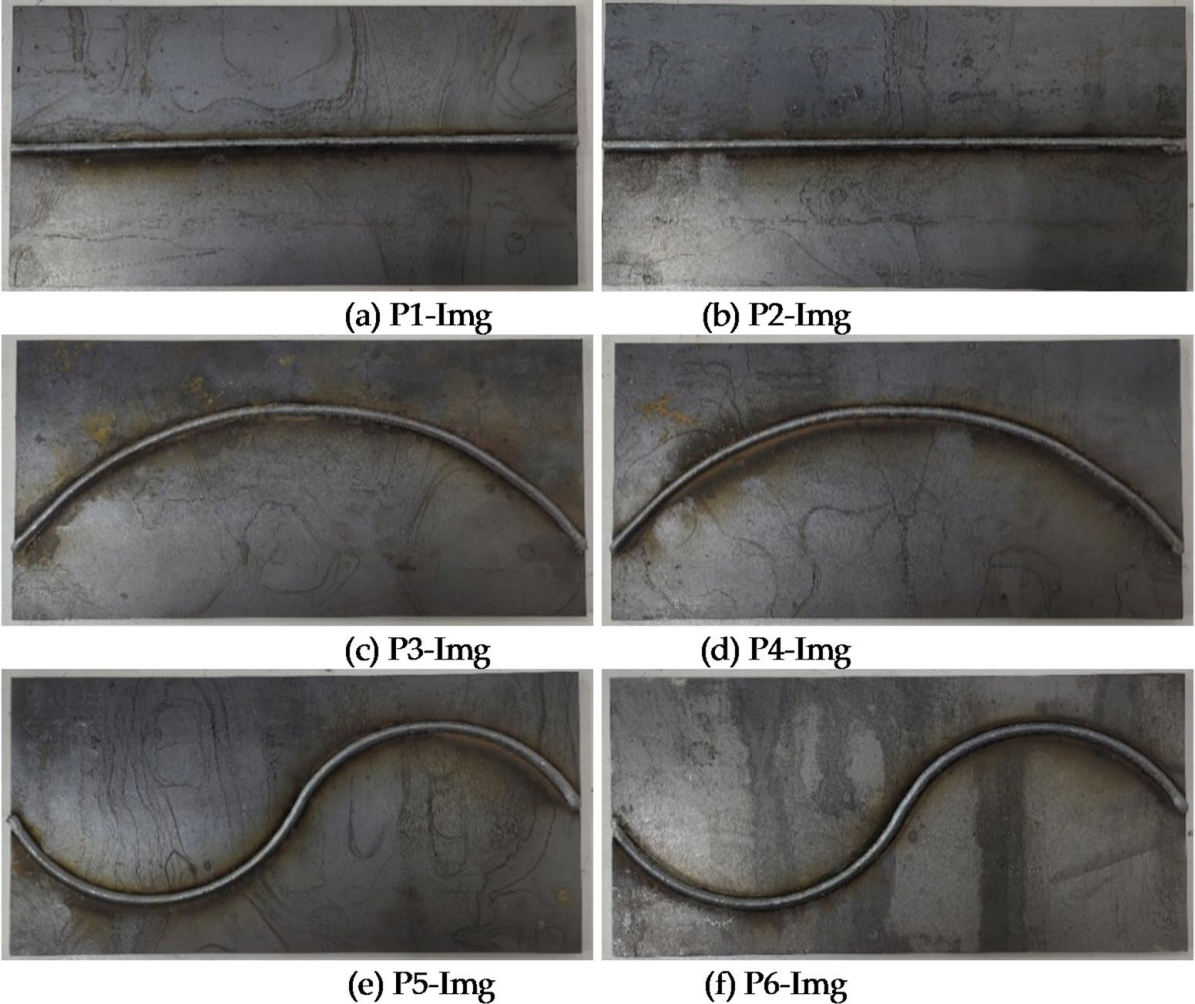
Real images of the six welded plates.

**Fig. 3:**
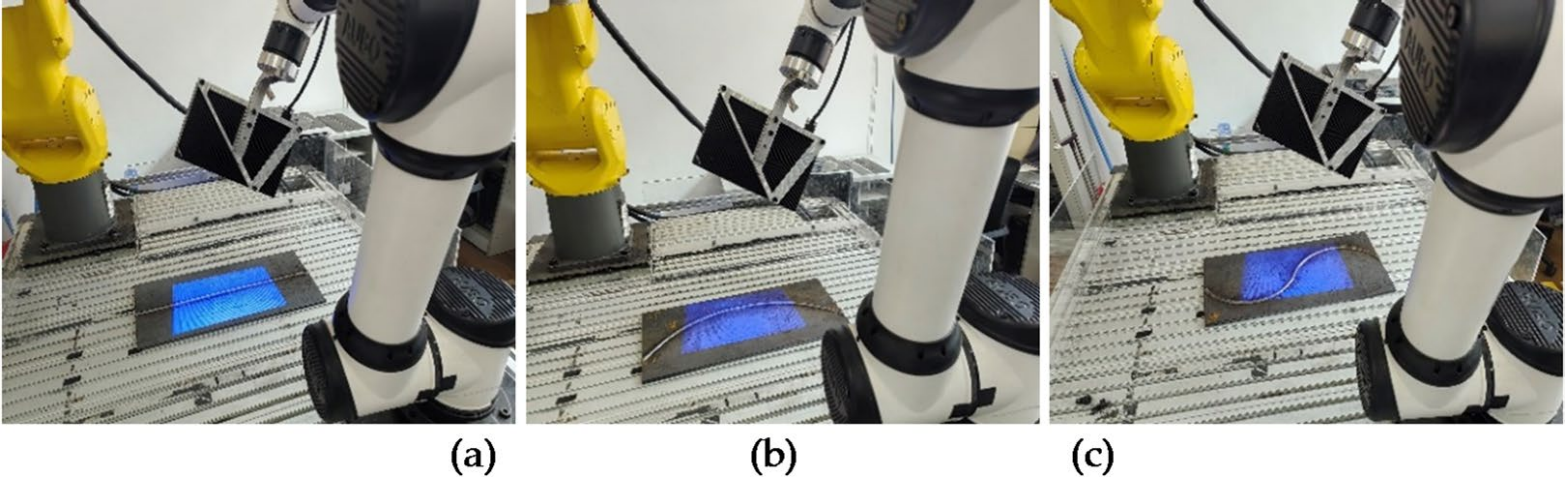
Representative real photographs of the data acquisition scenes for three typical weld morphologies: (a) straight-line weld, (b) curved-line weld, and (c) S-shaped weld.

The experimental data used in this study were collected from six independent welded plates, representing three typical weld morphologies: two straight-line welded plates, two curved-line welded plates, and two S-shaped welded plates. For each welded plate, the corresponding three-dimensional point cloud data of the real weld were acquired. Since different weld morphologies exhibit distinct characteristics in terms of geometric trajectory, local curvature variation, and boundary distribution, the inclusion of these three representative weld types enables a more comprehensive evaluation of the classification performance of the proposed method under different weld-shape conditions. Figure 2 presents the real images of the six welded plates. In addition, Fig. 3 shows representative photographs of the actual weld data acquisition scenes, illustrating the visual robotic platform and the experimental environment.

### Data acquisition and preprocessing

**Fig. 4:**
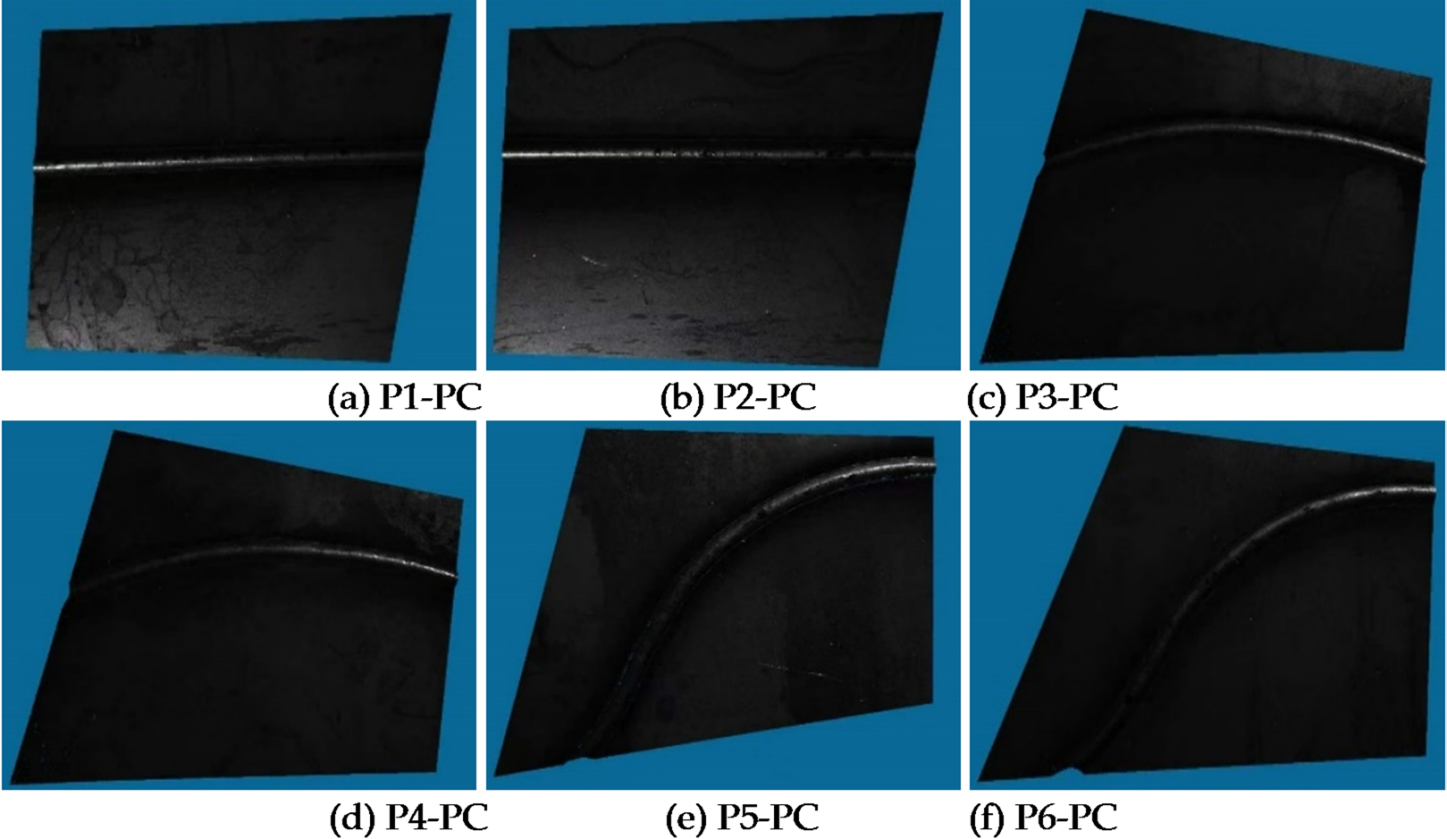
3D point clouds of the six welded plates visualized in CloudCompare: (a) P1-PC, (b) P2-PC, (c) P3-PC, (d) P4-PC, (e) P5-PC, and (f) P6-PC.

After obtaining the real images of the welded plates, three-dimensional point cloud data of the weld regions were further acquired. In this study, the depth camera was positioned 200 mm above the base-metal surface to capture the samples. The acquired data were processed using the software development kit (SDK) provided with the camera, which can simultaneously generate and display weld point clouds containing real 3D geometric information and RGB color information. The point cloud data were then imported into CloudCompare for visualization and subsequent processing, as shown in Fig. 4.

**Fig. 5:**
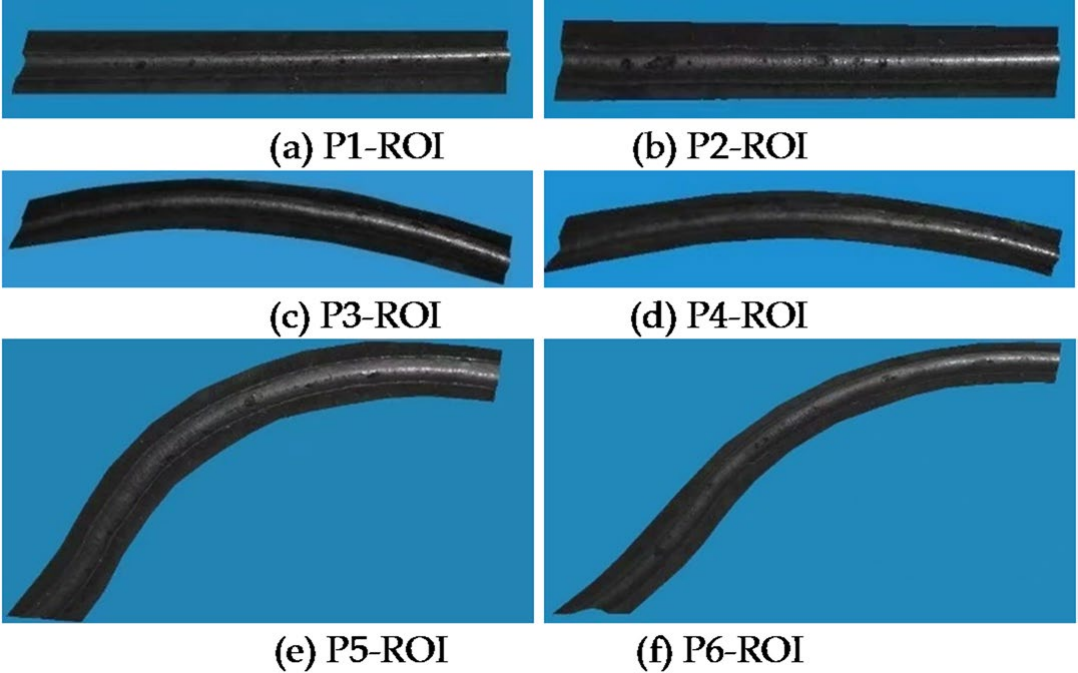
Preprocessed ROI regions of the six welded plates: (a) P1-ROI, (b) P2-ROI, (c) P3-ROI, (d) P4-ROI, (e) P5-ROI, and (f) P6-ROI.

The purpose of preprocessing was to extract the region of interest (ROI), suppress noise interference, reduce computational cost, and improve the accuracy and efficiency of the subsequent algorithms. First, a rapid cropping operation was performed to remove a large portion of the background base-metal region, thereby obtaining a preliminary ROI. Subsequently, a centroid-based voxel filtering method was applied within this region to further suppress noise and remove outliers while preserving the geometric characteristics of the weld surface. The preprocessed ROI regions are shown in Fig. 5. The extracted ROI regions were subsequently used for manual annotation and point-wise classification data construction. All photographs and visual materials shown in Figs. 1, 2, 3, 4 and 5 were taken, acquired, generated, or processed by the authors during the present study. No third-party copyrighted material is included in these figures.

### Calculation of geometric features

The point cloud acquired by the depth camera typically contains three-dimensional spatial coordinates and corresponding color information. To enhance the recognition capability of machine learning algorithms, local geometric features of the point cloud can be calculated based on the 3D coordinates. Geometric features are usually extracted from the covariance matrix constructed from a local neighborhood centered on a point. In a point cloud, the points within a given radius around a center point are called neighbors, and this neighborhood is also referred to as the support region. Geometric features are calculated based on the spatial relationships between this center point and its neighbors^30,31^.

For a given local support region, $P=(x,y,z)$ represents the current center point, and ${P_i}=({x_i},{y_i},{z_i})$represents the $i - th$ neighboring point of *P*, where *i* = 1,2,…,N. Here, N is the number of neighboring points in the local support region. The centroid of the neighborhood is denoted as $\bar {P}{\mathrm{=(}}\bar {x},\bar {y},\bar {z})$.The decentralized point-set matrix *Q* constructed from the local support region centered at point *P* can be expressed as:

1 $$Q=\left[ {\begin{array}{*{20}{c}} {{x_1} - \bar {x}}&{{y_1} - \bar {y}}&{{z_1} - \bar {z}} \\ \ldots & \ldots & \ldots \\ {{x_N} - \bar {x}}&{{y_N} - \bar {y}}&{{z_N} - \bar {z}} \end{array}} \right]$$

The covariance matrix *C* is calculated as:

2 $$C=\frac{1}{{N - 1}}{Q^T}Q$$

The eigenvalues of the covariance matrix are calculated and sorted in descending order as ${\lambda _1} \geqslant {\lambda _2} \geqslant {\lambda _3} \geqslant 0$. These eigenvalues correspond to the principal components of the local spatial distribution of the point cloud and can be used to calculate three-dimensional geometric descriptors. In addition, $\Lambda ={\lambda _1}+{\lambda _2}+{\lambda _3}$, denotes the sum of eigenvalues. In the umbrella curvature calculation, ${n_P}$ denotes the unit normal vector at the current point *P*. For the height-related feature, ${Z_i}$ denotes the reoriented height value of the $i - th$ neighboring point, and $\bar {Z}$ denotes the median of the ${Z_i}$ values. Based on these definitions, the geometric features used in this study are calculated as shown in Table 1.

The physical meanings of these geometric features in weld point-cloud recognition are as follows. Linearity describes whether the local neighborhood is distributed along a dominant direction, which is useful for characterizing elongated structures near weld boundaries. Planarity measures the degree to which the neighboring points lie on a local plane and is generally more prominent in relatively flat base-metal regions. Sphericity reflects the isotropic distribution of local points and may increase in locally scattered or irregular regions. Omnivariance describes the overall three-dimensional dispersion of the local neighborhood, while anisotropy measures the directional difference of the local point distribution. The sum of eigenvalues represents the total local geometric variation, and eigenentropy reflects the disorder or complexity of the local spatial distribution. Umbrella curvature describes the bending degree of the local surface around the current point and is therefore useful for capturing the convex bead surface and transition regions between the weld bead and the base metal. The median absolute deviation of the Z-coordinate, abbreviated as MAD, reflects the local height fluctuation after point-cloud reorientation and is closely related to the height difference between the weld bead and the base-metal surface.

**Table 1:** Calculation of geometric features.

Feature	Equation
Linearity	$({\lambda _1} - {\lambda _2})/{\lambda _1}$
Planarity	$({\lambda _2} - {\lambda _3})/{\lambda _1}$
Sphericity	${\lambda _3}/{\lambda _1}$
Omnivariance	$\sqrt[3]{{{\lambda _1}{\lambda _2}{\lambda _3}}}$
Anisotropy	$({\lambda _1} - {\lambda _3})/{\lambda _1}$
Sum of Eigenvalues	${\lambda _1}+{\lambda _2}+{\lambda _3}$
Eigenentropy	$- \sum\nolimits_{{i=1}}^{3} {\frac{{{\lambda _i}}}{\Lambda }} \log (\frac{{{\lambda _i}}}{\Lambda })$
Umbrella curvature	$\frac{{\mathrm{1}}}{N}\sum\limits_{{i=1}}^{N} {\left| {\frac{{({P_i} - P) \cdot {n_P}}}{{\left\| {{P_i} - P} \right\|}}} \right|}$
Median Absolute Deviation (MAD)	$median(|{Z_i} - \bar {Z}|)$

### Performance evaluation metrics

Overall Accuracy (OA), Precision, Recall, and the F1-Score are used as metrics to evaluate the research results. Accuracy represents the proportion of correctly predicted samples to all samples. $TP$ is the number of samples whose estimated label and ground truth are positive. $TN$ is the number of samples whose estimated label and ground truth are both negative. $FP$ is the number of samples whose estimated label is positive, but whose ground truth is negative. $FN$ is the number of samples whose estimated label is negative, but whose ground truth is positive^15^. The evaluation metrics are calculated as follows:

3 $$A{\mathrm{cc}}uracy=\frac{{TP+TN}}{{TP+FP+TN+FN}}$$

4 $$\ Precision=\frac{{TP}}{{TP+FP}}$$

5 $$\operatorname{Recall}=\frac{{TP}}{{TP+FN}}$$

6 $$F1\mathrm{-}Score = 2 \times \frac{\mathrm{Precision} \times \mathrm{Recall}}{\mathrm{Precision}+\mathrm{Recall}}$$

### SHAP

This study introduces the SHapley Additive exPlanations (SHAP) interpretability framework to comprehensively investigate geometric feature importance. Based on SHAP value calculations, this method offers a precise approach to interpreting the impact and contribution of each feature to the model^32^. The mechanism of the SHAP interpretability framework accounts for the cooperative relationships among different features, iteratively calculates the SHAP value for each feature, accurately assesses the influence of each feature on an individual prediction, and decomposes it into the contributions of various features^33^. The formula for calculating the SHAP value is as follows:

7 $${\phi _{\mathrm{i}}}=\sum\limits_{{A \subseteq \{ {x_1},...,{x_N}\} \backslash \{ {x_i}\} }} {\frac{{\left| A \right|!\left( {N - \left| A \right| - 1} \right)!}}{{N!}}} \left[ {{f_x}\left( {A \cup \{ {{\mathrm{x}}_i}\} } \right) - {f_x}\left( A \right)} \right]$$

In the formula: $\:{\phi\:}_{i}$ represents the Shapley value of the i-th feature; $\:N$ denotes the total number of features; $\:{f}_{x}\left(A\cup\:\left\{{x}_{i}\right\}\right)$ represents the model’s predicted value when feature $\:{x}_{i}$ is added based on feature subset $\:A$; and $\:{f}_{x}\left(A\right)$ represents the model’s predicted value when only feature subset $\:A$ is used.

## Machine learning models and optimization algorithms

### LightGBM

In this study, Light Gradient Boosting Machine (LightGBM) is an efficient machine learning method based on Gradient Boosting Decision Trees (GBDT). Renowned for its excellent predictive performance, high accuracy, and good interpretability, GBDT has become a widely used ensemble model in various machine learning tasks and has achieved leading results in numerous applications. LightGBM, proposed by Microsoft within the GBDT framework, aims to provide fast and accurate predictive solutions for large-scale, high-dimensional data and is regarded as a significant evolution of XGBoost. LightGBM introduces two key techniques: Gradient-based One-Side Sampling (GOSS) and Exclusive Feature Bundling (EFB)^34^. GOSS is an innovative sampling strategy that effectively reduces the number of data samples while maintaining the training accuracy of decision trees. EFB further enhances computational efficiency by efficiently merging mutually exclusive features to reduce feature dimensionality. Additionally, LightGBM employs a histogram-based decision tree algorithm, which discretizes continuous feature values into fixed-size histograms. The optimal split points are then quickly determined through histogram statistics, significantly accelerating the training speed. Regarding the tree growth strategy, LightGBM uses a leaf-wise growth strategy. It expands only the leaf with the highest information gain in the current layer, rather than growing all leaves level-wise uniformly. This approach helps improve model accuracy and mitigates overfitting^35^. This strategy, combined with a depth limit on leaf growth, helps improve predictive performance while reducing the risk of overfitting.

### Artificial Lemming algorithm

The Artificial Lemming Algorithm is a novel metaheuristic optimization algorithm introduced by Y. Xiao et al. in 2025^36^. Inspired by four typical behaviors of lemmings in nature—long-distance migration, burrowing, foraging, and predator avoidance—these behaviors are mathematically modeled to achieve a dynamic balance between global exploration and local exploitation. An energy-decreasing mechanism is incorporated to prevent premature convergence. This design enables the algorithm to effectively address common issues in traditional optimization methods, such as premature convergence, insufficient exploration capability, and poor robustness in high-dimensional, non-convex search spaces.

1. Exploration phase.

1) Long-distance Migration: This simulates the large-scale migratory behavior of lemmings during food shortages, designed to enhance the algorithm’s global exploration capability. The position update method is shown in Eq. (8):

8 $${\mathop Z\limits^{ \to } _i}(t+1)={\mathop Z\limits^{ \to } _{best}}(t)+F \times \overrightarrow {BM} \times (\mathop R\limits^{ \to } \times ({\mathop Z\limits^{ \to } _{best}}(t) - {\mathop Z\limits^{ \to } _i}(t)+(1 - \mathop R\limits^{ \to } ) \times ({\mathop Z\limits^{ \to } _i}(t) - {\mathop Z\limits^{ \to } _a}(t))$$

where ${\vec {Z}_i}(t)$ represents the current individual position, ${\mathop Z\limits^{ \to } _{best}}(t)$is the current optimal solution, ${\mathop Z\limits^{ \to } _a}(t)$ is a randomly selected individual, $\vec {R}$is a random vector within [− 1,1] simulating individual interaction, *F* is a direction flag used to dynamically adjust the search direction to avoid local optima, and $\overrightarrow {BM}$ is a Brownian Motion vector providing random step sizes to enhance exploration.

2) Digging Holes: This simulates lemmings digging burrows to seek shelter and store food, focusing on periodic exploration. The position update formula is given by Eq. (9):

9 $${\mathop Z\limits^{ \to } _i}(t+1)={\mathop Z\limits^{ \to } _i}(t)+F \times L \times (({\mathop Z\limits^{ \to } _{best}}(t) - {\mathop Z\limits^{ \to } _b}(t))$$

where ${\vec {Z}_b}(t)$ is a randomly selected individual to increase population diversity, and L is a random coefficient that varies with iterations, used to control the exploration intensity.

2. Exploitation phase.

1) Foraging for Food: This simulates lemmings randomly searching for food within a small range near their burrows, emphasizing local exploitation. The position update adopts a spiral encircling mechanism as shown in Eq. (10), where spiral is the spiral shape parameter:

10 $${\mathop Z\limits^{ \to } _i}(t+1)={\mathop Z\limits^{ \to } _{best}}(t)+F \times spiral \times rand \times {\mathop Z\limits^{ \to } _i}(t)$$

2) Escaping Predators: This simulates the process of lemmings fleeing back to their burrows when encountering danger, incorporating Lévy Flight to simulate evasive maneuvers. The position update formula is given by Eq. (11):

11 $${\mathop Z\limits^{ \to } _i}(t+1)={\mathop Z\limits^{ \to } _{best}}(t)+F \times G \times Levy(Dim) \times ({\mathop Z\limits^{ \to } _{best}}(t) - {\mathop Z\limits^{ \to } _i}(t))$$

where G is the escape coefficient, which decreases with iterations, indicating a diminishing escape capability. $Levy(Dim)$ is the $Levy$ Flight function, which generates large step sizes through a heavy-tailed distribution to help escape local optima.

3. Energy regulation mechanism.

In the ALA, the four aforementioned search strategies are closely linked to the energy level of an individual lemming. In the initial stages of the algorithm, lemmings are more inclined to perform global exploration to search for promising regions of the solution space. In the later stages of the search, they shift more towards local exploitation to more precisely approximate the global optimum. To achieve an effective balance between exploration and exploitation, an energy factor that decreases as the number of iterations increases is incorporated. When a lemming’s energy is high, its behavior is dominated by migration and digging holes, focusing on global exploration; when its energy is low, it switches to foraging and predator avoidance, emphasizing local exploitation. The calculation formula for this energy factor is given below as Eq. (12):

12 $$E(t)=4 \times \arctan [1 - \frac{t}{{{T_{\hbox{max} }}}}] \times \ln (\frac{1}{{rand}})$$

**Fig. 6:**
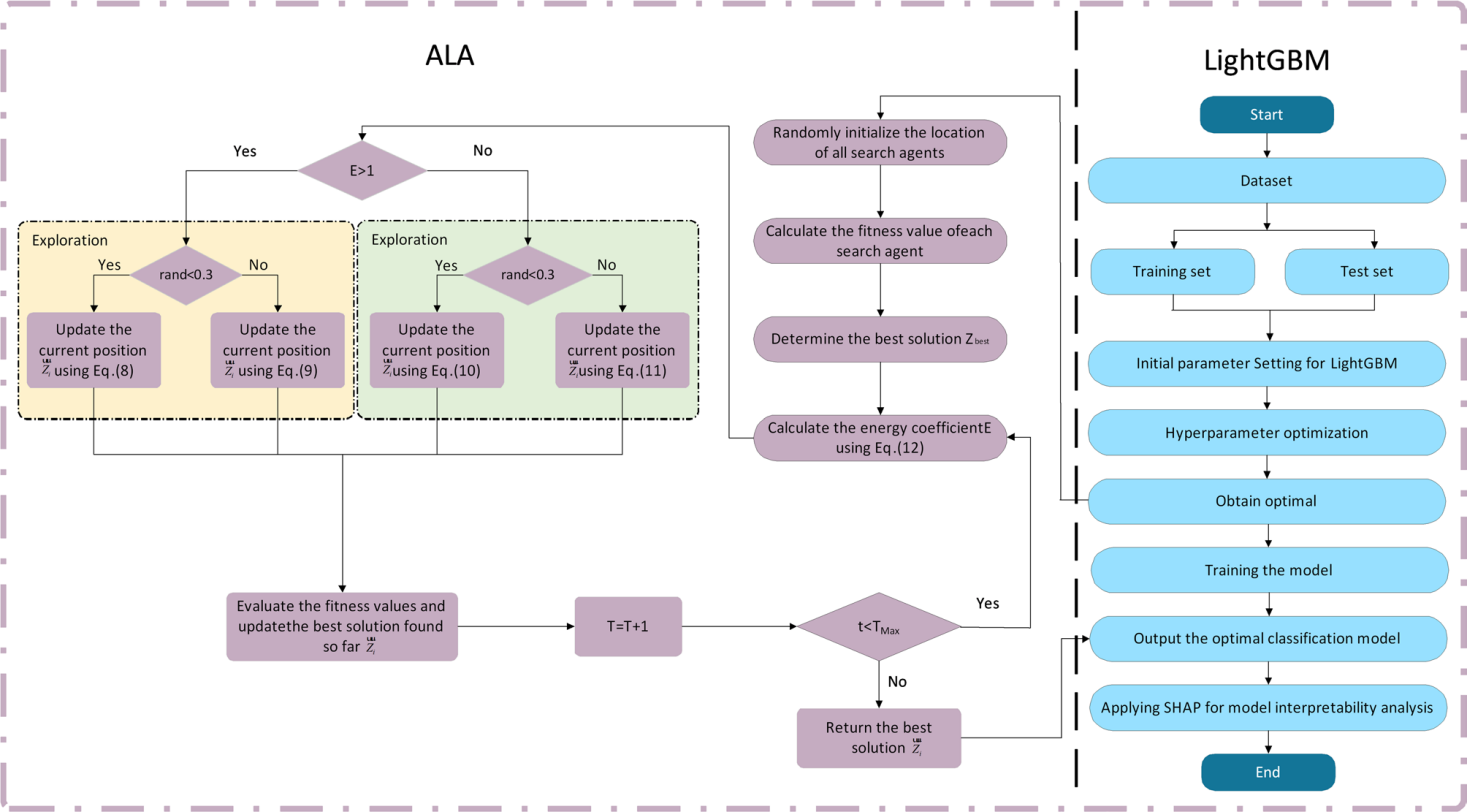
Framework of the ALA-LightGBM algorithm.

When $E(t)>1$, exploration behaviors (migration or digging holes) are executed; otherwise, exploitation behaviors (foraging or escaping) are performed. Here, $rand$ is a random number within (0, 1]. After reaching the maximum number of iterations ${T_{\hbox{max} }}$, the global optimal solution is output. The framework of the ALA-LightGBM algorithm is shown in Fig. 6.

### Starfish optimization algorithm

The Starfish Optimization Algorithm is derived from the biological characteristics and behavioral habits of starfish. This algorithm simulates the exploration, predation, and regeneration behaviors of starfish^37^. The SFOA consists of two main phases: exploration and exploitation. The exploration phase employs a hybrid search mode that combines five-dimensional and one-dimensional patterns, simulating the exploratory behavior of starfish. This improves computational efficiency and ensures search capability. The exploitation phase simulates the predation and regeneration behaviors of starfish, utilizing a bidirectional search strategy and a unique movement pattern. This enhances local optimization accuracy and convergence reliability, thereby effectively balancing exploration and exploitation during the search process.

1. Exploration phase.

1) When D > 5: The five-dimensional search mode is employed. First, it is necessary to acquire the current position of the starfish and the range explored by the starfish, as shown in Eq. (13):

13 $$\{ \begin{array}{*{20}{c}} {Y_{{i,p}}^{T}=X_{{i,p}}^{T}+{a_1}(X_{{best,p}}^{T} - X_{{i,p}}^{T})\cos \theta ,r \leqslant 0.5} \\ {Y_{{i,p}}^{T}=X_{{i,p}}^{T} - {a_1}(X_{{best,p}}^{T} - X_{{i,p}}^{T})\sin \theta ,r>0.5} \end{array}$$

where $Y_{{{\mathrm{i}},p}}^{T}$ and $X_{{i,p}}^{T}$ represent the acquired position and the current position of the starfish on the p-th dimension, respectively, $X_{{best,p}}^{T}$ is the best position on the p-th dimension, *p* denotes five randomly selected dimensions from the *D* dimensions, where *r* is a random number between (0,1), ${a_1}$ is randomly generated for the update position of each candidate in each iteration, and $\theta$ varies with the number of iterations.

2) When D ≤ 5: The one-dimensional search mode is employed (updating only one dimension), simulating the fine-grained exploration where a starfish moves only a single arm. The updated position can be established as:

14 $$Y_{{i,p}}^{T}={E_t}X_{{i,p}}^{T}+{A_1}(X_{{k1,p}}^{T} - X_{{i,p}}^{T})+{A_2}(X_{{k2,p}}^{T} - X_{{i,p}}^{T})$$

where $X_{{{K_1},p}}^{T}$ and $X_{{{K_2},p}}^{T}$ represent the positions on the p-th dimension of two randomly selected starfish, respectively, ${A_1}$ and ${A_2}$ are two random numbers between (-1, 1), *p* is a randomly selected number within the *D* dimensions, and ${E_t}$ is the starfish energy.

2. Exploitation phase.

1) Predation behavior (primary update strategy) The update rule for each starfish in predation behavior is modeled as:

15 $$Y_{i}^{T}=X_{i}^{T}+{r_1}{d_{m1}}+{r_2}{d_{m2}}$$

where ${r_1}$ and ${r_2}$ are random numbers between (0, 1), ${d_{m1}}$ and ${d_{m2}}$ are randomly selected from the top ${d_m}$ solutions. The Starfish Optimization Algorithm employs a parallel bidirectional search strategy. In each iteration, a portion of the candidate solutions are guided to move towards the direction of the current better solutions for local exploitation; meanwhile, the remaining candidate solutions are intentionally guided in the opposite direction for exploration. This mechanism effectively maintains population diversity and enhances the algorithm’s ability to escape local optima.

**Fig. 7:**
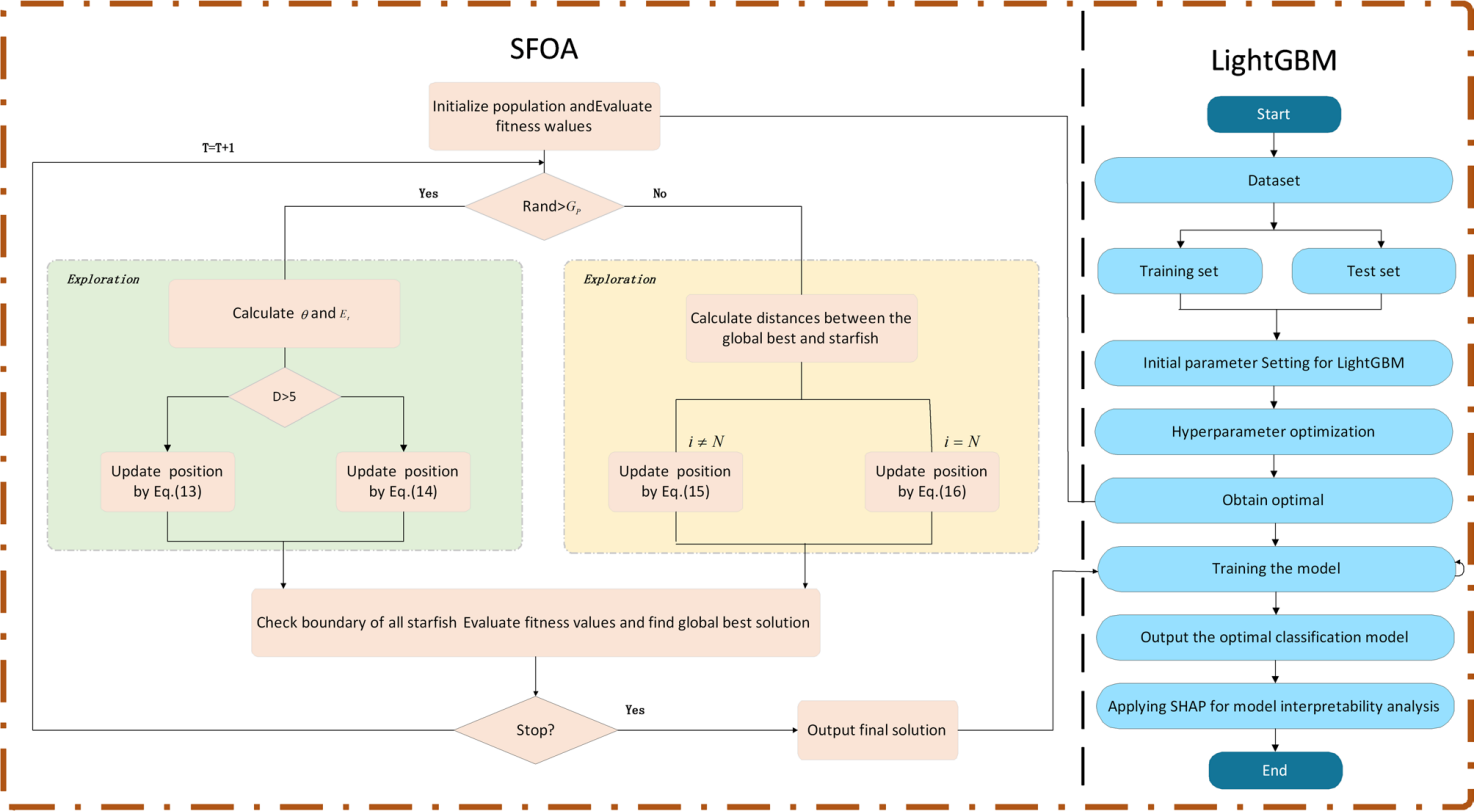
Framework of the SFOA-LightGBM algorithm.

2) Regeneration behavior (applicable only to the lowest-ranked individual in the population) Due to their slow movement during predation, starfish are vulnerable to attacks by other predators. If captured by a predator, a starfish may autotomize and lose an arm to escape. Therefore, the regeneration phase in the SFOA is implemented only for the lowest-ranked starfish in the population ($i=N$). Since the regeneration process takes several months in their lifecycle, the starfish moves extremely slowly. Consequently, the update rule is modeled for the regeneration phase, and the position is updated as follows:

16 $$Y_{i}^{T}=\exp ( - T \times N/{T_{\hbox{max} }})X_{i}^{T}$$

where *T* denotes the current iteration, ${T_{\hbox{max} }}$ represents the maximum number of iterations, and *N* is the population size. The framework of the SFOA-LightGBM algorithm is illustrated in Fig. 7.

### Alpha evolution algorithm

The Alpha Evolutionary Algorithm (AE) is a recently proposed metaheuristic optimization method inspired by the adaptive step-size updating mechanism of the Alpha operator^38^. The key innovation of AE lies in the design of the Alpha operator, which integrates multiple information extraction and utilization strategies. Specifically, it combines base-vector adaptation, random step-size mechanisms, and adaptive step-size adjustment to enhance the search capability of the algorithm.

Through this mechanism, AE achieves an effective balance between exploration and exploitation, thereby improving the overall optimization performance. The general framework of the AE algorithm mainly consists of four stages: population initialization, Alpha operator update, boundary constraint handling, and selection strategy.

1. Alpha operator.

The Alpha operator is the core of AE, used to update solutions. Its mathematical model is:

17 $$E_{{\mathrm{i}}}^{{t+1}}=P+\alpha \Delta {{\mathrm{r}}_i}+\theta \cdot ({W_i}+E_{i}^{t} - P - {L_i})$$

where $E_{i}^{{t+1}}$ represents the $i - th$ solution to be evolved in generation $t+1$, *P* is the adaptive base vector that determines the starting point of evolution, $\alpha \Delta {r_i}$ denotes the random step size, which provides global exploration capability, $\theta \cdot ({W_i}+E_{i}^{t} - P - {L_i})$ is the adaptive step size, which provides local exploitation capability, ${W_i}$ and ${L_i}$ are solutions sampled from the candidate matrix X, satisfying $f({W_i}) \leqslant f({E_i}) \leqslant f({L_i})$, and $\theta$ is the control parameter.

1) Adaptive Base Vector *P*

*P* is computed in two ways, and an evolutionary path is introduced to enhance intergenerational correlation. The base vector is calculated as follows:

18 $$P=\left\{ {\begin{array}{*{20}{c}} {diagonal(A),{\text{ }}if{\text{ }}rand(0,1)<0.5} \\ {{\text{ }}\omega {\text{B, otherwise }}} \end{array}} \right.$$

$diagonal(A)$ represents a function, which is a D-th order square matrix, obtained by sampling with replacement from the candidate solutions multiplied by D. Here, *B* is a matrix with K rows and D columns, which is obtained by sampling without replacement. Evolution path adaptation:

19 $$P=\left\{ {\begin{array}{*{20}{c}} {{c_a} \times P_{a}^{t}+(1 - {c_a}) \times diagonal(A)=P_{a}^{{t+1}},{\text{ }}if{\text{ }}rand(0,1)<0.5} \\ {{\text{ }}{c_b} \times P_{b}^{t}+(1 - {c_b}) \times {\omega _b}=P_{b}^{{t+1}}{\text{, otherwise }}} \end{array}} \right.$$

In the equation, ${c_a}$ and ${c_b}$ are learning rates (which decrease with iteration), ${P_a}$ and ${P_b}$ respectively record the historical paths of the two types of basis vectors, preventing them from getting stuck in local optima.

2) Random Step Size $\alpha \Delta {r_i}$

For the random step size $\alpha \Delta {r_i}$, this component provides a global search capability. The attenuation factor is a nonlinearly decreasing value, calculated using Eq. (20):

20 $$\alpha ={e^{\left( {\ln \left( {\frac{{MaxFEs - FEs}}{{MaxFEs}}} \right) - {{\left( {\frac{{4FEs}}{{MaxFEs}}} \right)}^2}} \right)}}$$

Herein, $FEs$ denotes the fitness evaluations, and $MaxFEs$ represents the maximum number of fitness evaluations; *e* denotes the exponential function, and $\ln$ denotes the natural logarithm function.

${\Delta _r}$ is defined as a decaying perturbation under the influence of time delay, calculated by Eq. (21):

21 $$\Delta r=(ub - lb) \cdot (2{R_1} \cdot {R_2} - {R_2}) \cdot S$$

Here, ${R_1}$ and ${R_2}$represent random real matrices generated by rand(0,1,[N, D]), used to produce perturbations, while *S* denotes a random integer matrix containing only 0 or 1, generated by randi (0,1,[N, D]), which makes trade-offs based on dimensions.

2. Boundary constraint.

To ensure that the solution remains within the feasible region, AE employs the “half-distance” method. When a solution exceeds the boundary, it is reset to the midpoint of the boundary, balancing the efficiency of constraint handling.

22 $$E_{i}^{j}=\left\{ {\begin{array}{*{20}{c}} {\frac{{{E_{i,j}}+ub}}{2},{\text{ }}if{\text{ }}{{\mathrm{E}}_{i,j}}>ub} \\ {\frac{{{E_{i,j}}+lb}}{2},{\text{ }}if{\text{ }}{{\mathrm{E}}_{i,j}}<lb} \\ {{E_{i,j}},{\text{ }}otherwise{\text{ }}} \end{array}} \right.$$

3. Selection strategy.

**Fig. 8:**
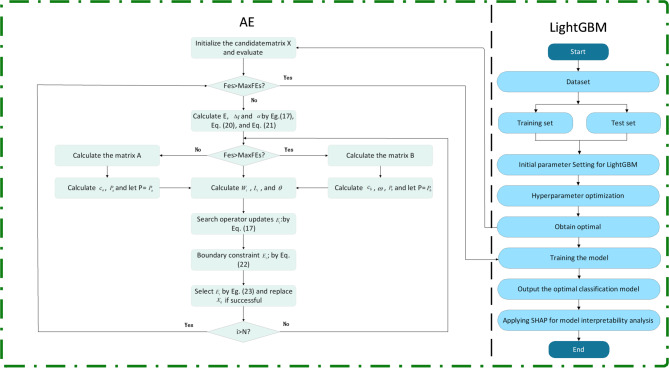
Framework of the AE-LightGBM algorithm.

A selection strategy is a method for incorporating relevant solutions into the next-generation set. Common selection strategies include greedy selection, roulette wheel selection, tournament selection, truncation selection, and others. Among these, greedy selection passes successfully evolved solutions to the next generation through a straightforward method without requiring additional operations. Therefore, this selection strategy is introduced into the AE algorithm, and its model can be represented by Eq. (23). Figure 8 illustrates the framework of the AE-LightGBM algorithm.

23 $$X_{k}^{{t+1}}=\left\{ {\begin{array}{*{20}{c}} {E_{i}^{{t+1}},{\text{ }}if{\text{ }}f\left( {E_{i}^{{t+1}}} \right){\text{ <=f}}\left( {E_{i}^{t}} \right){\text{ }}} \\ {X_{k}^{t},{\text{ }}otherwise{\text{ }}} \end{array}} \right.$$

The three optimizers used in this study differ in their mechanisms for balancing exploration and exploitation. ALA adjusts the search process through lemming-inspired behavior switching, including migration, digging, foraging, and predator avoidance. SFOA relies on starfish-inspired exploration, predation, and regeneration behaviors, with an emphasis on bidirectional movement and population diversity. AE updates candidate solutions through the Alpha operator, which combines adaptive base-vector construction, random perturbation, and adaptive step-size adjustment. These different search mechanisms provide complementary strategies for LightGBM hyperparameter optimization and form the basis for the comparative evaluation in the following experiments.

## Results and discussion

In this study, the influence of different neighborhood scales on machine learning models was determined, the enhancement of model performance by intelligent optimization algorithms was verified, and the importance of geometric features was analyzed using the SHAP interpretable framework. The first step of this research involved experiments with different support radius values to determine the most suitable neighborhood search range, with specified radii of 0.5 mm, 1 mm, 1.5 mm, and 2 mm. Three ensemble machine learning algorithms—RF, XGBoost, and LightGBM—were used to classify the 3D point cloud of the weld seam, aiming to identify the optimal combination of neighborhood scale and machine learning model. The second step aimed to improve the classification performance of the selected machine learning model by employing the Artificial Lemming Algorithm, Alpha Evolution Algorithm, and Starfish Optimization Algorithm. The third step involved model interpretability analysis based on the SHAP framework. The best-performing AE-LightGBM model was chosen, and its decision-making mechanism was interpreted using the SHAP framework to quantitatively analyze the contribution and influence of different geometric features on the classification results of the weld seam point cloud.

### Experimental data split and evaluation protocol

**Fig. 9:**
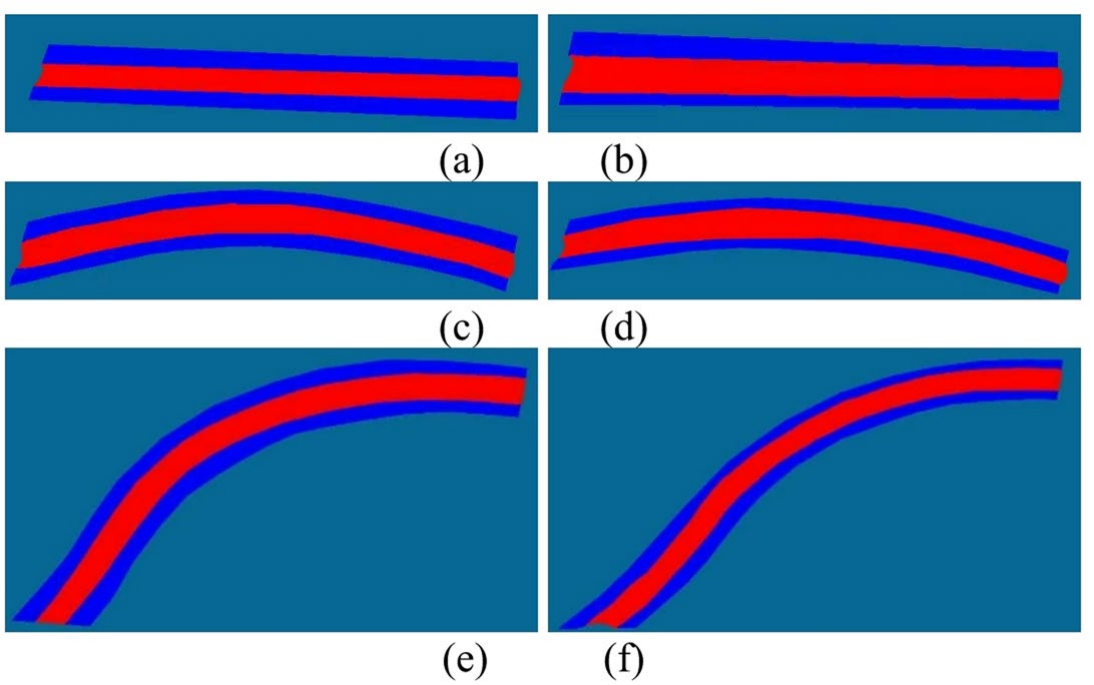
Annotated ROI regions used for binary point-wise classification: (a) P1-AnnROI, (b) P2-AnnROI, (c) P3-AnnROI, (d) P4-AnnROI, (e) P5-AnnROI, and (f) P6-AnnROI.

After ROI extraction, the weld-region point clouds were manually annotated using CloudCompare software^24^. During annotation, each point within the ROI was assigned to one of two classes, namely weld bead or base metal, based on the geometric boundary information of the weld surface and with reference to the corresponding real images of the welded plates. In this way, binary class labels for supervised learning were established. An example of the annotated ROI is shown in Fig. 9, where different colors are used to distinguish the two classes.

To ensure a rigorous evaluation and reduce the risk of data leakage, a welded-plate-level independent split strategy was adopted. The split unit was the welded plate rather than individual points, ROIs, or overlapping local neighborhoods. The six welded plates were denoted as P1–P6, where P1–P2, P3–P4, and P5–P6 corresponded to straight-line, curved-line, and S-shaped welds, respectively. For each weld morphology, one welded plate was used for training and the other welded plate was reserved for testing, resulting in a 1:1 training/test split at the welded-plate level, as summarized in Table 2.

ROI extraction, annotation, and labeled-point construction were conducted independently within each welded plate. All samples generated from a welded plate remained in the subset to which that welded plate was assigned. Therefore, local neighborhoods may overlap within the same subset, but they do not cross the training/test boundary because the training and test data were obtained from different welded plates. Hyperparameter tuning was performed only on the training set using five-fold cross-validation, and the independent test plates were used only for final evaluation.

Accuracy, Precision, Recall, and F1-Score were adopted as the evaluation metrics to comprehensively assess the performance of the model in the binary point-wise classification task of weld bead versus base metal. Since the training and test data were collected from different welded plates, the experimental results can provide a more realistic estimate of model performance on unseen welded-plate data.

**Table 2:** Weld morphology-specific training/test split at the welded-plate level.

Weld morphology	Training plate	Test plate	Training points	Test points	Train/test ratio
Straight-line	P1	P2	422,000	422,310	1:1
Curved-line	P3	P4	401,872	402,310	1:1
S-shaped	P5	P6	412,238	421,210	1:1

### Model comparison and optimal support domain

To reduce reliance on empirically selected three-dimensional neighborhood structures, different neighborhood radii were investigated to determine a suitable support range for geometric feature extraction. Specifically, four radius values, namely 0.5 mm, 1.0 mm, 1.5 mm, and 2.0 mm, were evaluated. The neighborhood radius determines the spatial range used to construct local geometric descriptors, and therefore directly affects the balance between local detail preservation and neighborhood stability.

**Fig. 10:**
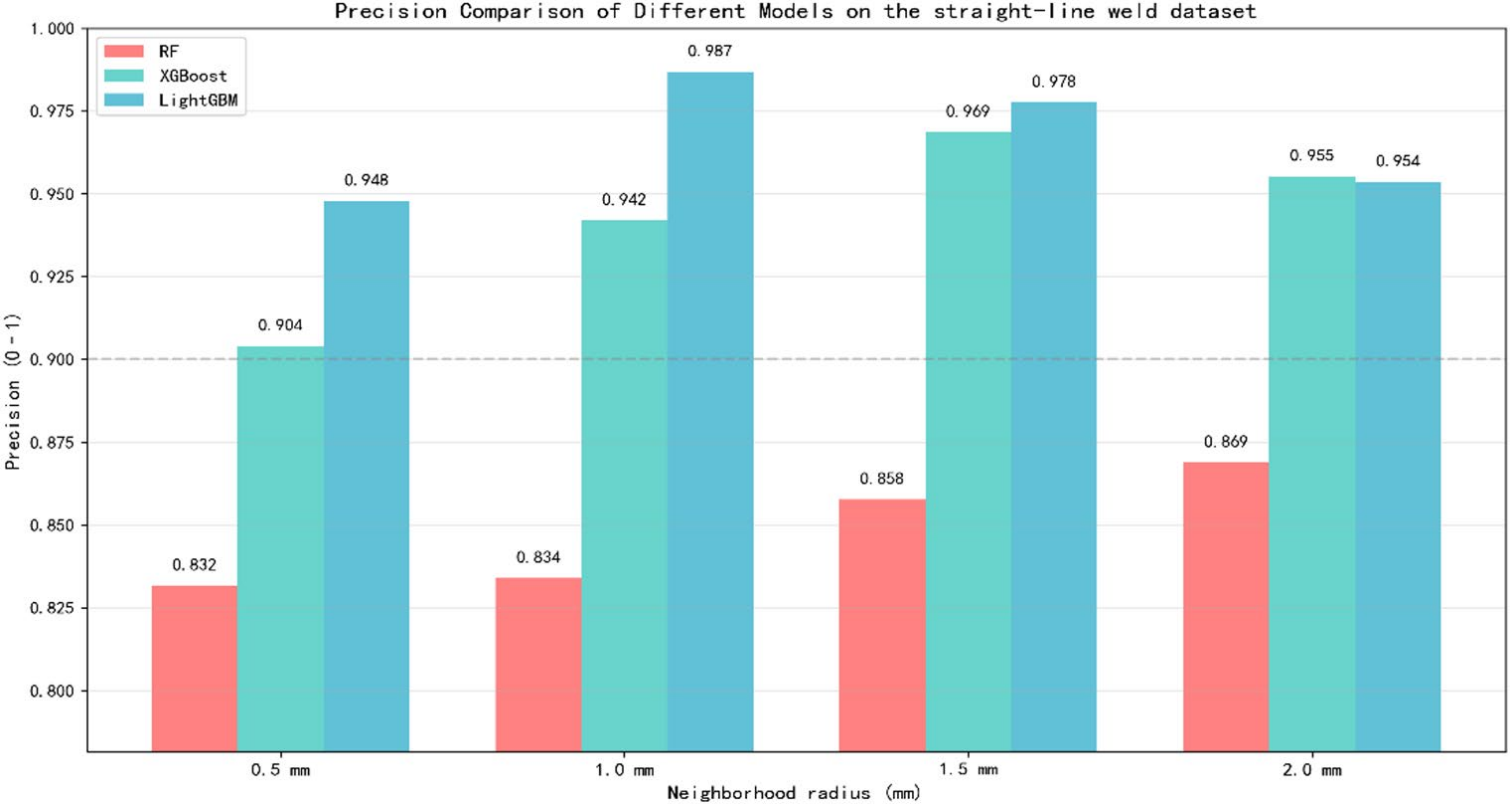
Precision of baseline classifiers under different neighborhood radii for straight-line welds.

**Fig. 11:**
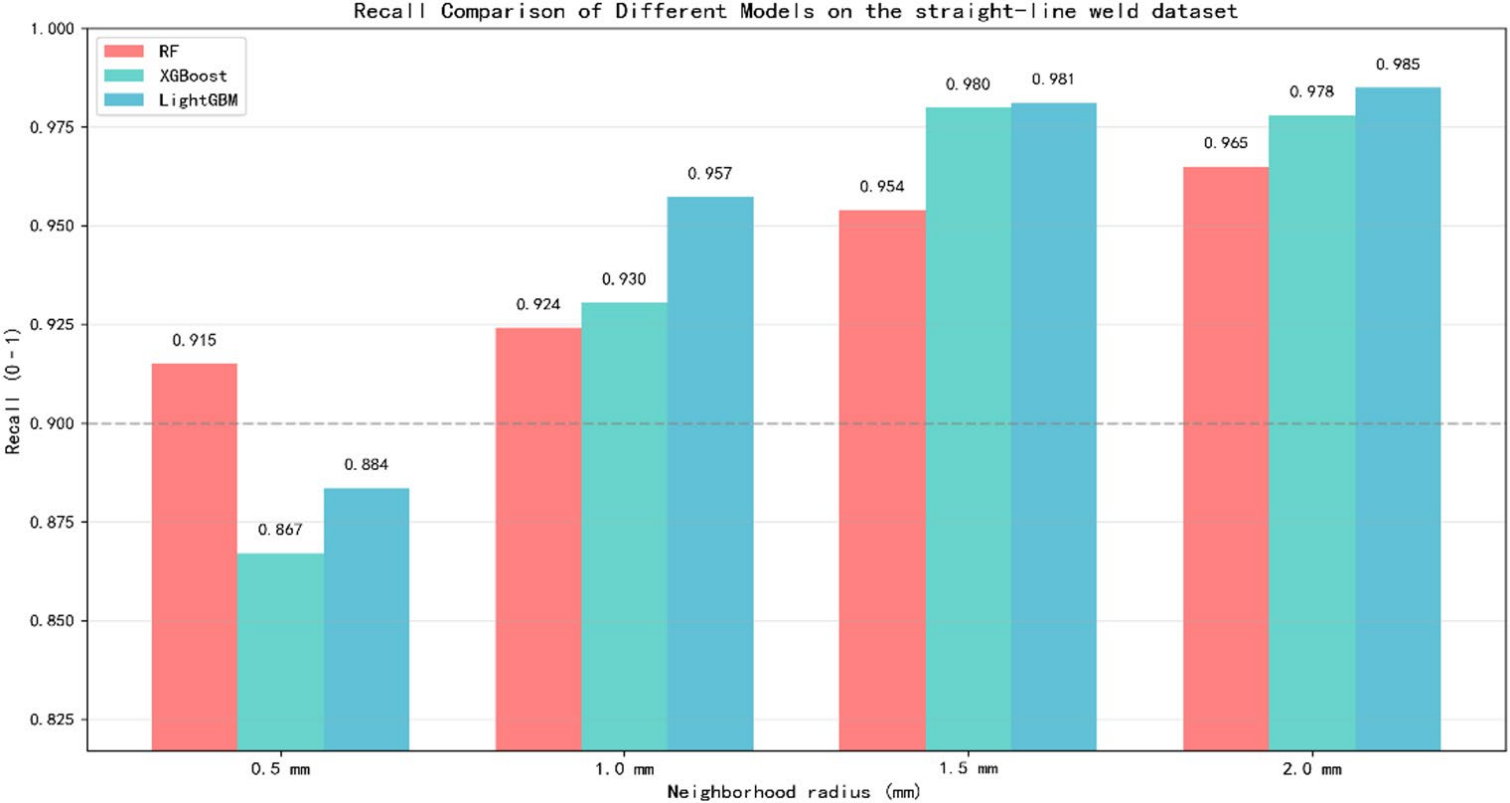
Recall of baseline classifiers under different neighborhood radii for straight-line welds.

**Fig. 12:**
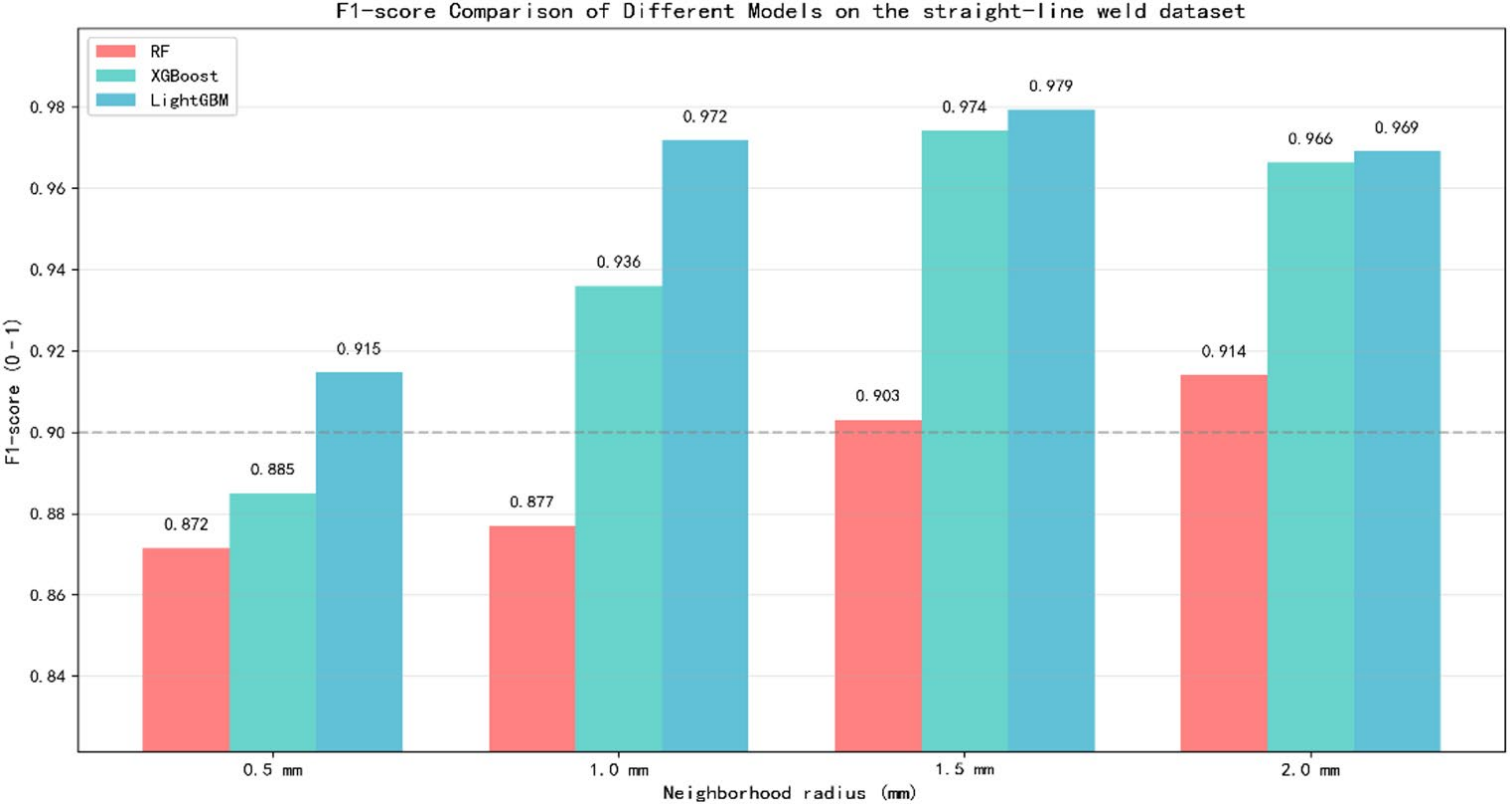
F1-Score of baseline classifiers under different neighborhood radii for straight-line welds.

**Fig. 13:**
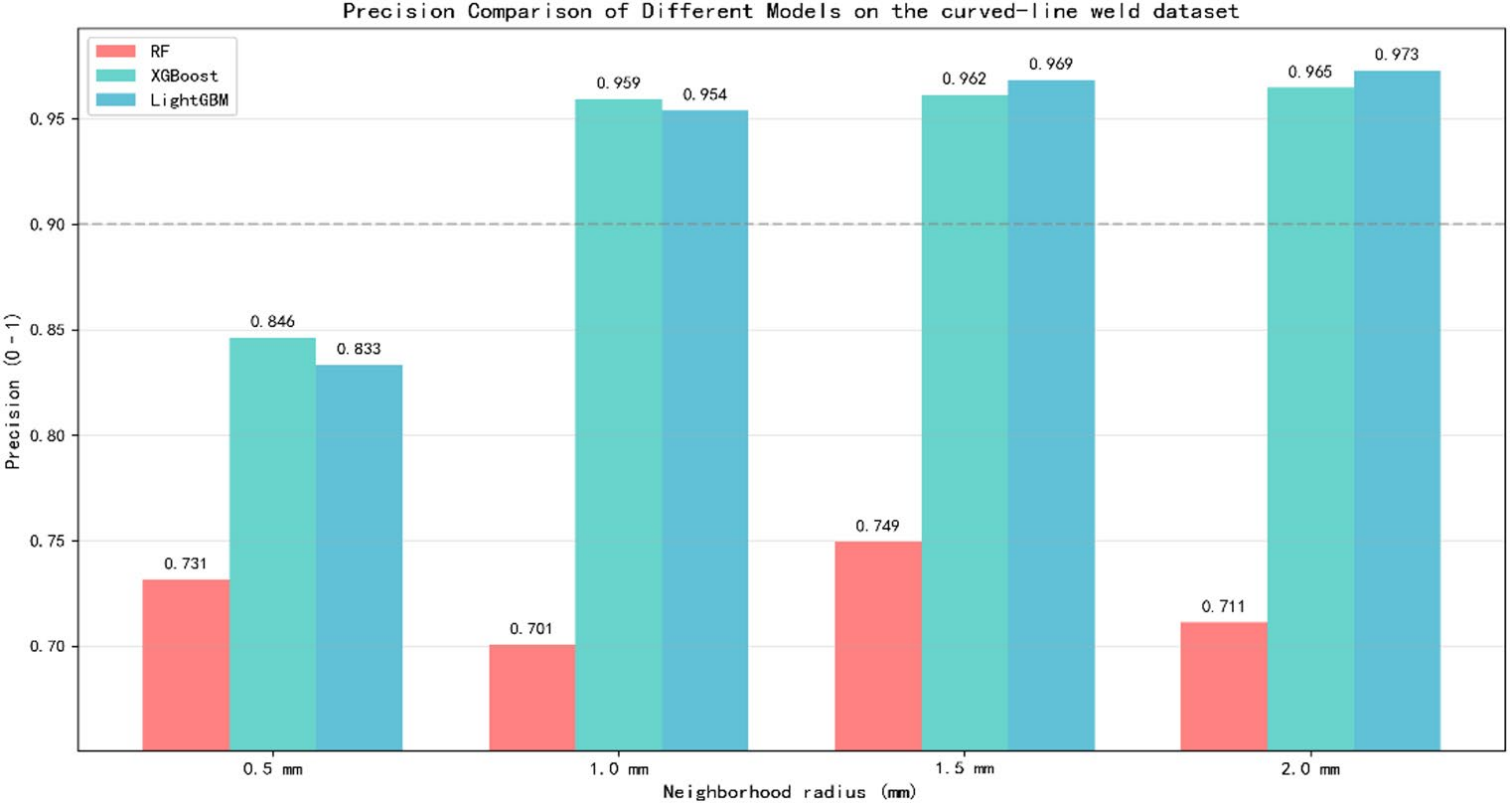
Precision of baseline classifiers under different neighborhood radii for curved-line welds.

**Fig. 14:**
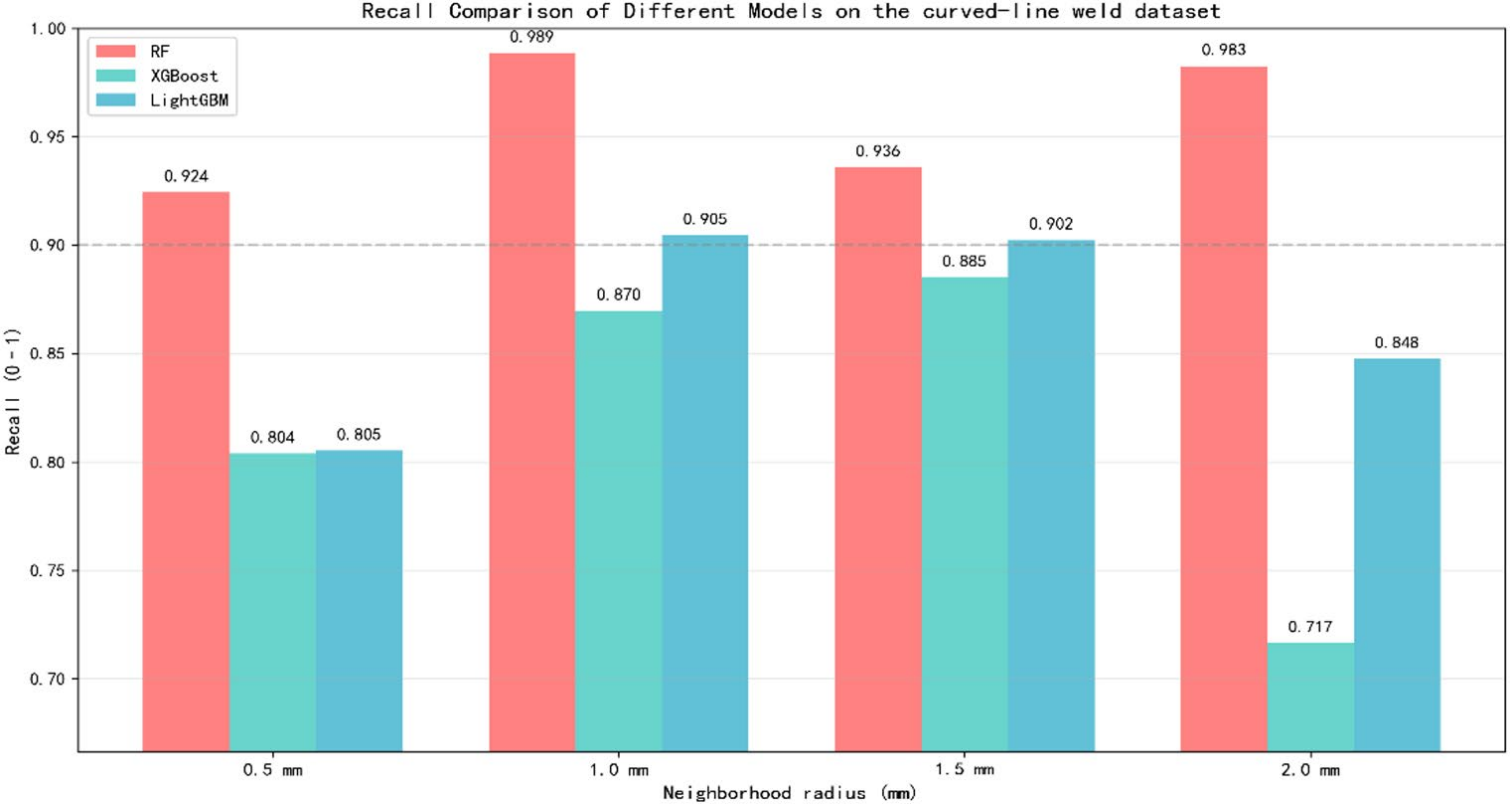
Recall of baseline classifiers under different neighborhood radii for curved-line welds.

**Fig. 15:**
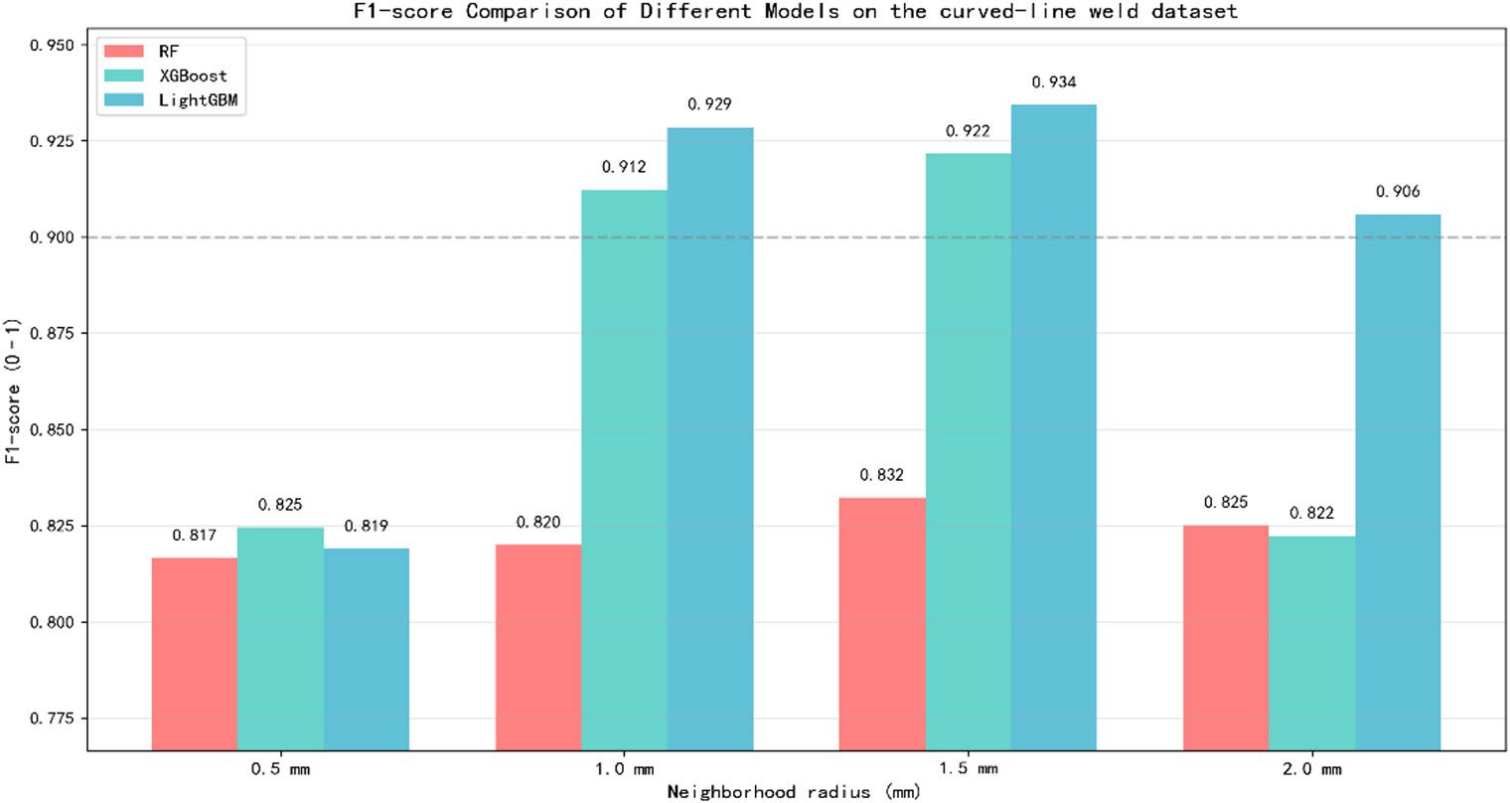
F1-Score of baseline classifiers under different neighborhood radii for curved-line welds.

**Fig. 16:**
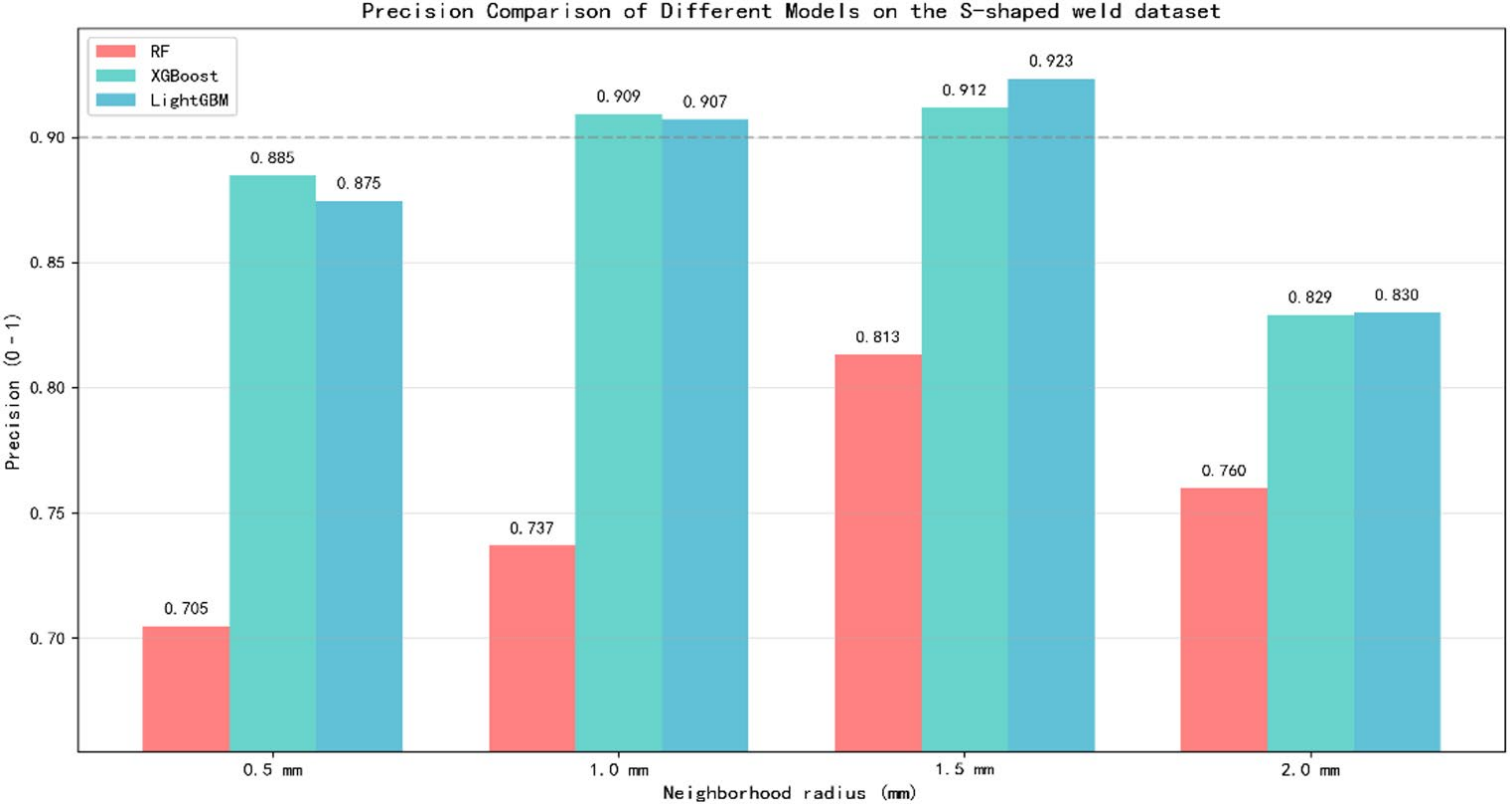
Precision of baseline classifiers under different neighborhood radii for S-shaped welds.

**Fig. 17:**
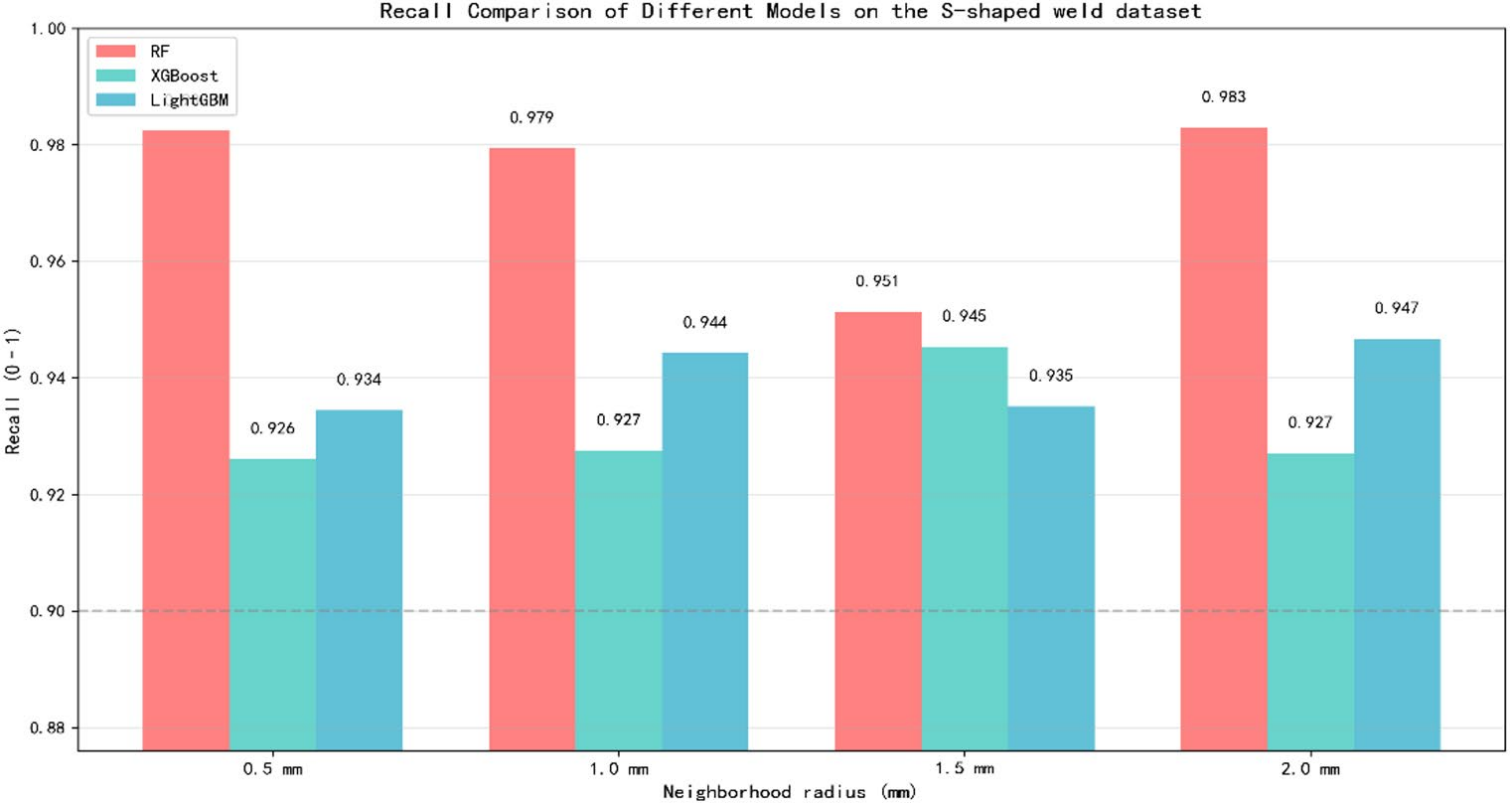
Recall of baseline classifiers under different neighborhood radii for S-shaped welds.

**Fig. 18:**
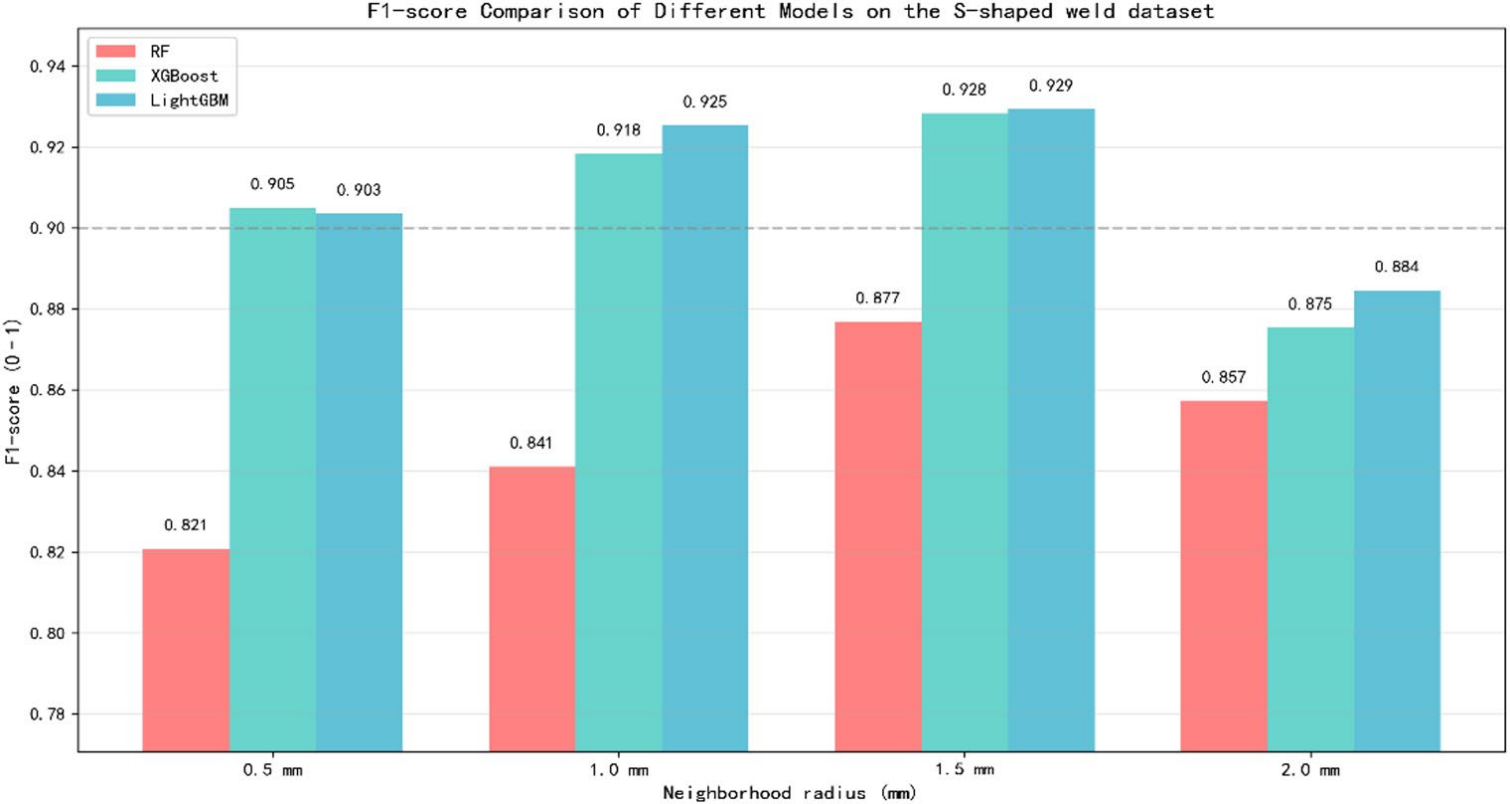
F1-Score of baseline classifiers under different neighborhood radii for S-shaped welds.

The OA results under different neighborhood radii are summarized in Table 3. Precision, Recall, and F1-Score are further presented in Figs. 10, 11, 12, 13, 14, 15, 16, 17 and 18 to analyze the classification behavior of different models in greater detail. These three metrics are dimensionless and range from 0 to 1. In Figs. 10, 11, 12, 13, 14, 15, 16, 17 and 18, the horizontal axis represents the neighborhood radius in millimeters, and the vertical axis represents the corresponding metric value. Since the metric values are relatively close in several cases, the vertical axes are displayed using enlarged local ranges rather than the full 0–1 scale to make the differences among models more visible.

As shown in Table 3, when the neighborhood radius was set to 1.5 mm, the OA values of the models generally reached their best levels across the three weld morphologies, and LightGBM achieved the highest OA values among the compared classifiers. Therefore, the 1.5 mm neighborhood radius was selected for the subsequent hyperparameter optimization experiments.

**Table 3:** OA values of baseline classifiers under different neighborhood radii.

Classifier	Dataset	0.5 mm	1.0 mm	1.5 mm	2.0 mm
RF	Straight-line	0.8633	0.8498	0.9050	0.8924
	Curved-line	0.8278	0.8199	0.8435	0.8273
	S-shaped	0.7708	0.8022	0.8571	0.8250
XGBoost	Straight-line	0.9070	0.9564	0.9737	0.9657
	Curved-line	0.8581	0.9306	0.9376	0.8716
	S-shaped	0.8961	0.9118	0.9219	0.8588
LightGBM	Straight-line	0.9165	0.9719	0.9781	0.9682
	Curved-line	0.8523	0.9423	0.9473	0.9270
	S-shaped	0.8933	0.9186	0.9239	0.8679

On the straight-line weld dataset, performance analysis was conducted based on overall accuracy (Table 3), precision (Fig. 10), recall (Fig. 11), and the F1-Score (Fig. 12), with the following results: The LightGBM model achieved the highest OA value (0.9781) at a 1.5 mm neighborhood. Its precision performed best at 1 mm and 1.5 mm neighborhoods, with values of 0.987 and 0.978, respectively. Recall continuously improved as the neighborhood size increased, reaching optimal levels at 1.5 mm and 2 mm, with values of 0.981 and 0.985, respectively. The F1-Score was also optimal at 1 mm and 1.5 mm, with values of 0.972 and 0.979. Considering all metrics comprehensively, LightGBM demonstrated the best overall performance at the 1.5 mm neighborhood. The XGBoost model exhibited relatively balanced overall performance, with its performance gradually improving as the neighborhood size increased, peaking at 1.5 mm and slightly declining at 2 mm. However, all its metrics were slightly lower than those of LightGBM. The Random Forest (RF) model showed acceptable performance in terms of recall, indicating its effectiveness in capturing positive samples. However, its precision and F1-Score were significantly lower than those of LightGBM and XGBoost, and its accuracy was also notably inferior. This suggests that the RF model tends to detect more positive cases, but also produces a relatively larger number of false positives.

On the curved-line weld dataset, performance analysis was conducted based on overall accuracy (Table 3), precision (Fig. 13), recall (Fig. 14), and the F1-Score (Fig. 15), with the following results: The LightGBM model achieved the best comprehensive performance in terms of accuracy, precision, recall, and F1-Score at the 1.5 mm neighborhood. At the 2 mm neighborhood, although its precision was slightly better than that at 1.5 mm, its recall and F1-Score significantly decreased, indicating that while expanding the neighborhood improved prediction certainty, it compromised overall coverage and balance. The XGBoost model exhibited stable overall performance, gradually improving as the neighborhood size increased, peaking at 1.5 mm. At 2 mm, although its precision increased slightly, its recall and F1-Score also showed a clear decline, and its overall performance remained slightly inferior to that of LightGBM. The RF model performed notably well in recall, especially reaching 0.989 at the 1 mm neighborhood. However, its precision and F1-Score were only 0.701 and 0.820, respectively, and its accuracy was only 0.8278. This indicates that, although the RF model can effectively identify positive cases, it also yields a relatively large number of false positives under the present evaluation setting.

On the S-shaped dataset, an analysis was conducted based on overall accuracy (Table 3), precision (Fig. 16), recall (Fig. 17), and the F1-Score (Fig. 18), with the following results: The LightGBM model achieved the highest accuracy (0.9239) at the 1.5 mm neighborhood. Its precision was optimal at the 1 mm and 1.5 mm neighborhoods, with values of 0.907 and 0.923, respectively; recall values were 0.944 and 0.935, respectively; and F1-Scores were 0.925 and 0.929, respectively. Considering all metrics comprehensively, the 1.5 mm neighborhood is the optimal choice. The XGBoost model demonstrated balanced overall performance, improving gradually as the neighborhood size increased, peaking at 1.5 mm and declining at 2 mm. Its OA, precision, recall, and F1-Score at the 1.5 mm neighborhood were 0.9219, 0.912, 0.945, and 0.928, respectively, overall slightly lower than those of LightGBM. The RF model still showed high recall, reaching 0.983 at 2 mm, but its precision and F1-Score were only 0.76 and 0.857, respectively, and its accuracy was only 0.825. This indicates that the model has a high number of misclassifications in the S-shaped weld classification task, suggesting relatively weaker adaptability to this weld morphology under the present setting. In summary, LightGBM with the 1.5 mm neighborhood offers the best overall performance and is the optimal choice for this dataset.

### Optimization algorithm comparison and efficiency evaluation

To reduce the influence of randomness in metaheuristic optimization and ensure a fair comparison, AE, ALA, and SFOA were evaluated under a unified experimental protocol. According to the results in section “Model comparison and optimal support domain”, the neighborhood radius was fixed at 1.5 mm, and LightGBM was adopted as the common baseline classifier in all optimization experiments. The three optimizers shared the same hyperparameter search space and optimization settings. Specifically, the population size was set to 15, the maximum number of iterations was set to 20, and the total number of fitness evaluations was fixed at 300. The stopping criterion was defined as reaching the maximum number of fitness evaluations. The optimization objective was OA, and the validation performance was estimated using five-fold cross-validation on the training set. To improve statistical reliability, each optimizer was independently repeated 15 times with different random seeds.

The hyperparameter optimization focused on key LightGBM parameters that directly affect model complexity and classification performance, namely n_estimators, learning_rate, max_depth, and num_leaves. Among them, n_estimators denotes the number of boosting trees, learning_rate is a dimensionless shrinkage coefficient used in each boosting iteration, max_depth controls the maximum depth of each decision tree, and num_leaves denotes the maximum number of leaves in a tree. These parameters are algorithmic hyperparameters and do not have physical units. To ensure comparability, AE, ALA, and SFOA searched within the same predefined parameter ranges. The detailed search space and the representative optimal hyperparameter settings obtained by the three optimizers are listed in Table 4. Although the final parameter configurations differ across optimizers, all results were obtained under identical search constraints and optimization budgets, which provides a fair basis for comparison.

**Table 4:** Hyperparameter search ranges and representative optimal settings for AE, ALA, and SFOA.

Dataset	Parameter	Search space	AE-LightGBM	ALA-LightGBM	SFOA-LightGBM
			Optimal hyperparameters	Optimal hyperparameters	Optimal hyperparameters
Straight-line	n_estimators	(50, 300)	298	288	287
	learning_rate	(0.01, 0.2)	0.081	0.09	0.09
	max_depth	(1, 12)	9	8	8
	num_leaves	(20, 100)	87	77	78
Curved-line	n_estimators	(50, 300)	211	233	212
	learning_rate	(0.01,0.2)	0.112	0.09	0.1
	max_depth	(1, 12)	7	8	8
	num_leaves	(20, 100)	55	61	64
S-shaped	n_estimators	(50, 300)	189	177	181
	learning_rate	(0.01, 0.2)	0.166	0.1	0.1
	max_depth	(1, 12)	10	9	10
	num_leaves	(20, 100)	98	88	95

The repeated-run classification results are summarized in Table 5 in the form of mean ± standard deviation over 15 independent runs. Among the three optimized models, AE-LightGBM achieved the most favorable overall performance on the straight-line, curved-line, and S-shaped test subsets. More importantly, its advantage was consistently observed across repeated runs rather than arising from an isolated favorable trial. Since the present task is a binary point-wise classification problem of weld bead versus base metal, the reported Accuracy, Precision, Recall, and F1-Score collectively indicate that AE-LightGBM provides a better overall balance between classification correctness, positive-class precision, and recall under the current evaluation protocol.

Compared with the original LightGBM model, all three optimized variants improved the classification performance on the morphology-specific test subsets to different extents. Among them, AE-LightGBM showed the most favorable overall performance across the three test subsets, indicating that metaheuristic hyperparameter optimization is beneficial for improving the classification effectiveness of LightGBM in the current weld point-cloud task. Although the degree of improvement varies across different weld morphologies and evaluation metrics, the overall trend remains consistent, and AE delivers the most stable performance gain under the present evaluation setting.

**Table 5:** Mean ± standard deviation of the classification performance over 15 independent runs on the three morphology-specific test subsets.

Dataset	Classifier	Precision (0–1)	Recall (0–1)	F1-Score (0–1)	OA (0–1)
Straight-line	AE-LightGBM	0.9870 ± 0.0014	0.9884 ± 0.0013	0.9877 ± 0.0012	0.9876 ± 0.0012
	ALA-LightGBM	0.9783 ± 0.0025	0.9812 ± 0.0023	0.9797 ± 0.0023	0.9795 ± 0.0022
	SFOA-LightGBM	0.9778 ± 0.0027	0.9815 ± 0.0025	0.9796 ± 0.0024	0.9794 ± 0.0024
Curved-line	AE-LightGBM	0.9502 ± 0.0036	0.9580 ± 0.0033	0.9540 ± 0.0034	0.9622 ± 0.0031
	ALA-LightGBM	0.9438 ± 0.0043	0.9573 ± 0.0040	0.9505 ± 0.0041	0.9592 ± 0.0038
	SFOA-LightGBM	0.9438 ± 0.0045	0.9579 ± 0.0042	0.9510 ± 0.0042	0.9595 ± 0.0039
S-shaped	AE-LightGBM	0.9402 ± 0.0051	0.9509 ± 0.0048	0.9455 ± 0.0049	0.9405 ± 0.0046
	ALA-LightGBM	0.9355 ± 0.0057	0.9498 ± 0.0053	0.9426 ± 0.0054	0.9376 ± 0.0050
	SFOA-LightGBM	0.9350 ± 0.0059	0.9430 ± 0.0055	0.9389 ± 0.0056	0.9378 ± 0.0051

**Fig. 19:**
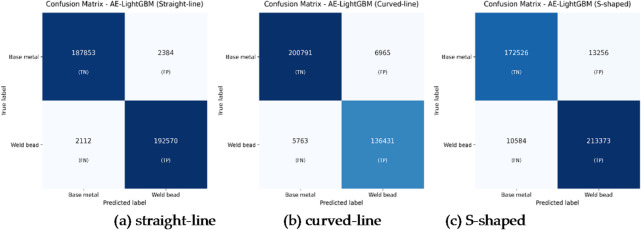
Representative confusion matrices of AE-LightGBM on the three morphology-specific test subsets for the binary point-wise classification task: (a) straight-line, (b) curved-line, and (c) S-shaped.

To complement the repeated-run statistical results, Fig. 19 presents representative confusion matrices of AE-LightGBM on the straight-line, curved-line, and S-shaped test subsets. As shown in Fig. 19, the predicted results in all three subsets are consistently organized under the same binary label setting, which further clarifies that the present task is a binary point-wise classification problem of weld bead versus base metal rather than a weld-morphology classification task. Meanwhile, the confusion matrices provide a more intuitive view of the distributions of true positives, true negatives, false positives, and false negatives for the final optimized model. It should be noted that the quantitative performance averaged over repeated runs is reported in Table 5, whereas Fig. 19 is included here mainly to provide a clearer visualization of the binary classification outcome on the three morphology-specific test subsets. In addition, the confusion matrices in Fig. 19 were calculated using the valid test samples after neighborhood-based geometric feature extraction.

To further examine whether the observed performance differences are statistically meaningful, a nonparametric significance analysis was conducted on the repeated-run OA results. Specifically, the Wilcoxon signed-rank test with Holm correction was applied, and the results are reported in Table 6. The comparisons show that the differences between AE-LightGBM and ALA-LightGBM, as well as between AE-LightGBM and SFOA-LightGBM, are statistically significant under the current setting, whereas the difference between ALA-LightGBM and SFOA-LightGBM is not statistically significant. These results strengthen the conclusion that the performance advantage of AE-LightGBM is statistically supported under the unified evaluation protocol.

**Table 6:** Wilcoxon signed-rank test with Holm correction for OA over 15 independent runs.

Comparison	W statistic	Raw *p*-value	Holm-corrected *p*-value	Significant (α = 0.05)	Better performer
ALA vs. AE	9.0	0.002014	0.004028	Yes	AE
ALA vs. SFOA	36.0	0.187622	0.187622	No	—
AE vs. SFOA	8.0	0.001526	0.004028	Yes	AE

**Fig. 20:**
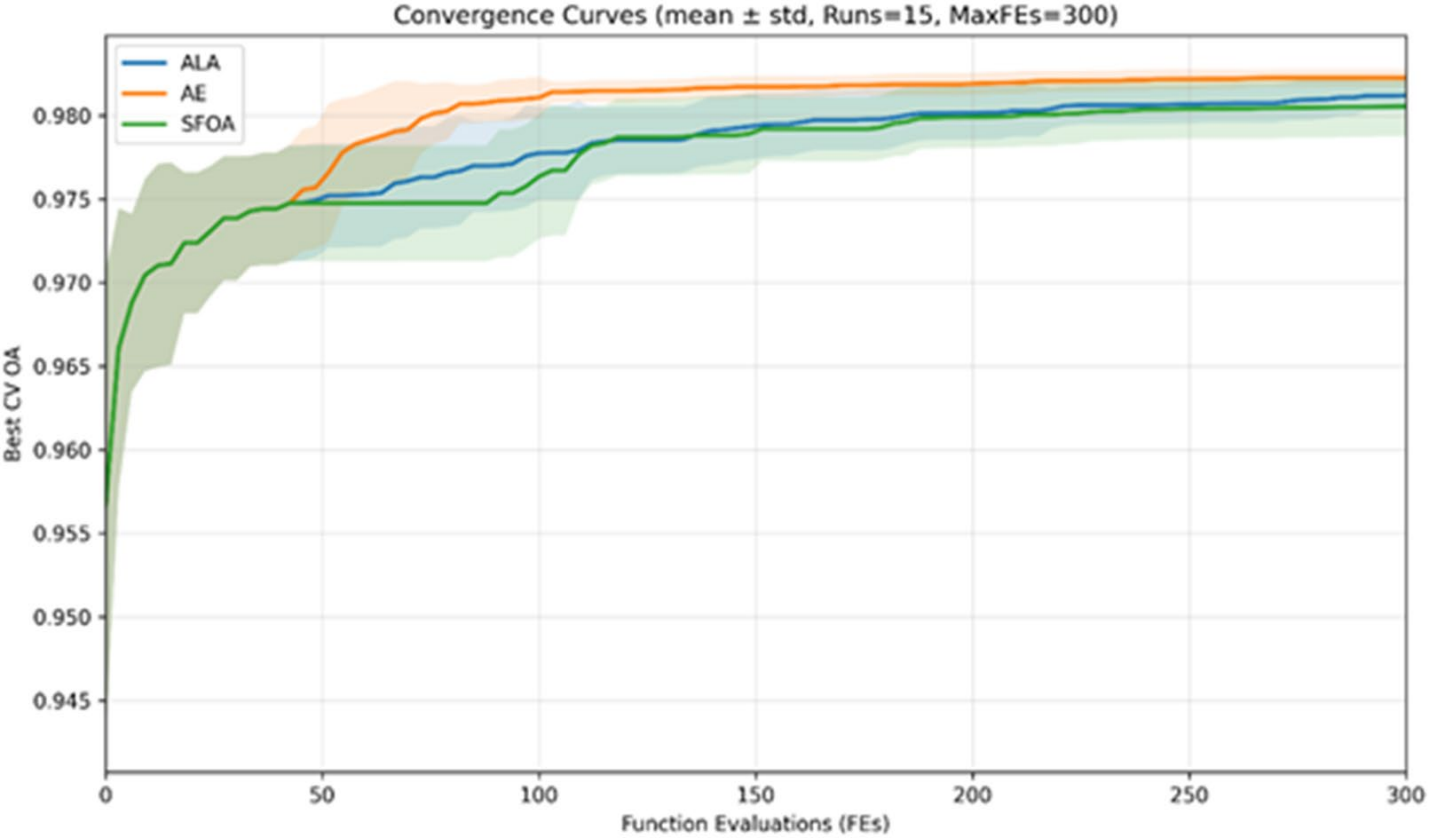
Mean ± standard deviation convergence curves of AE, ALA, and SFOA over 15 independent runs under the unified optimization budget.

The convergence behavior of the three optimizers was further analyzed to assess optimization efficiency and stability beyond the final scores. As shown in Fig. 20, the convergence curves describe the fitness evolution with respect to the number of fitness evaluations. Compared with ALA and SFOA, AE exhibits a more favorable optimization trajectory, with faster performance improvement in the early stage and more stable convergence in the later stage. This suggests that AE achieves a better balance between exploration and exploitation during the hyperparameter search process. Therefore, the convergence analysis is consistent with the repeated-run statistical results and further supports the effectiveness of AE as a hyperparameter optimizer for the current task.

Considering the practical deployment requirements in robotic and industrial scenarios, computational efficiency was also evaluated in terms of hyperparameter tuning time, model training time, inference latency, throughput, and peak memory usage. The corresponding results are summarized in Table 7. The results show that AE-LightGBM not only yields the best overall classification performance, but also maintains competitive computational cost under the current implementation environment. Although the three optimizers exhibit different characteristics in individual efficiency metrics, AE-LightGBM provides a more favorable overall trade-off between predictive performance and computational overhead. This is particularly important for real-world weld recognition applications, where deployment feasibility depends on both classification accuracy and computational efficiency.

All experiments and efficiency measurements were conducted under the same hardware and software environment to ensure comparability. The host computer was equipped with an AMD Ryzen 7 5800 × 8-core processor, 32 GB RAM, and an NVIDIA GeForce RTX 3080 GPU, running the Windows 10 operating system. The software implementation was developed in PyCharm 2023. Under this unified evaluation setting, AE-LightGBM achieves the most favorable overall balance between classification performance and computational cost among the compared methods. Therefore, AE-LightGBM can be regarded as a promising approach for the current weld point-cloud classification task.

**Table 7:** Efficiency comparison in terms of hyperparameter tuning time, training time, inference latency, throughput, and peak memory usage.

Optimizer	Tuning time (s)	Training time (s)	Inference latency (ms/sample)	Throughput (samples/s)	Peak memory (MB)
ALA	973.32 ± 129.99	4.644 ± 0.583	0.000610 ± 0.000070	1,658,311 ± 170,298	643.69 ± 20.29
AE	917.27 ± 215.84	4.757 ± 0.579	0.000644 ± 0.000081	1,573,405 ± 168,822	652.17 ± 23.14
SFOA	920.45 ± 150.03	4.252 ± 0.965	0.000586 ± 0.000144	1,804,030 ± 435,365	644.95 ± 20.34

### Geometric feature importance analysis

To improve the interpretability of the optimized AE-LightGBM model, this section analyzes the relative contributions of geometric features to the classification results by combining feature-importance ranking and SHAP-based explanation. Since AE-LightGBM achieved the best overall performance across the three morphology-specific datasets, it was selected as the target model for interpretability analysis. The purpose of this section is to provide an interpretable view of how different geometric descriptors influence the binary point-wise classification of weld bead versus base metal.

In the feature-importance plots, the horizontal axis represents the feature importance score calculated by AE-LightGBM. In the SHAP summary plots, each point corresponds to one sample, and the color represents the feature value of that sample. Red indicates a higher feature value, whereas blue indicates a lower feature value. The horizontal position of each point represents its SHAP value. A positive SHAP value indicates that the feature increases the model output toward the weld-bead class, whereas a negative SHAP value indicates that the feature increases the model output toward the base-metal class.

**Fig. 21:**
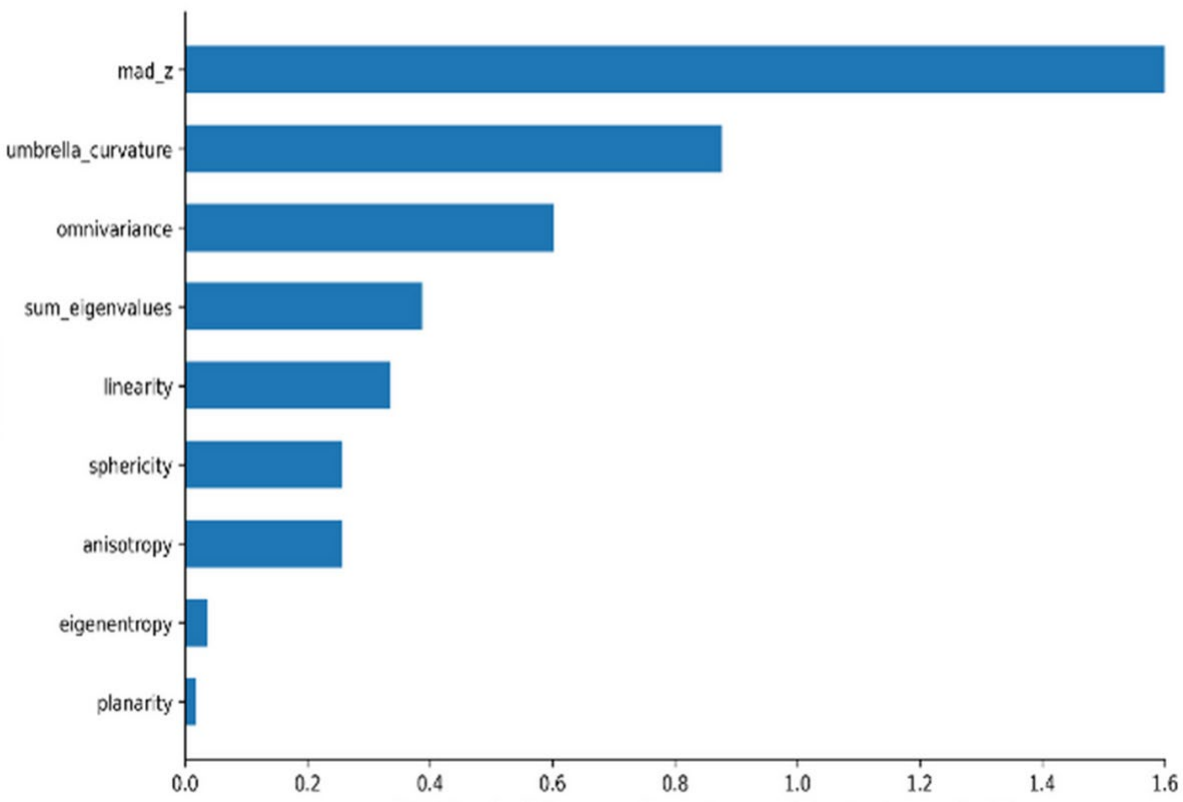
Geometric feature importance ranking for the straight-line weld dataset.

**Fig. 22:**
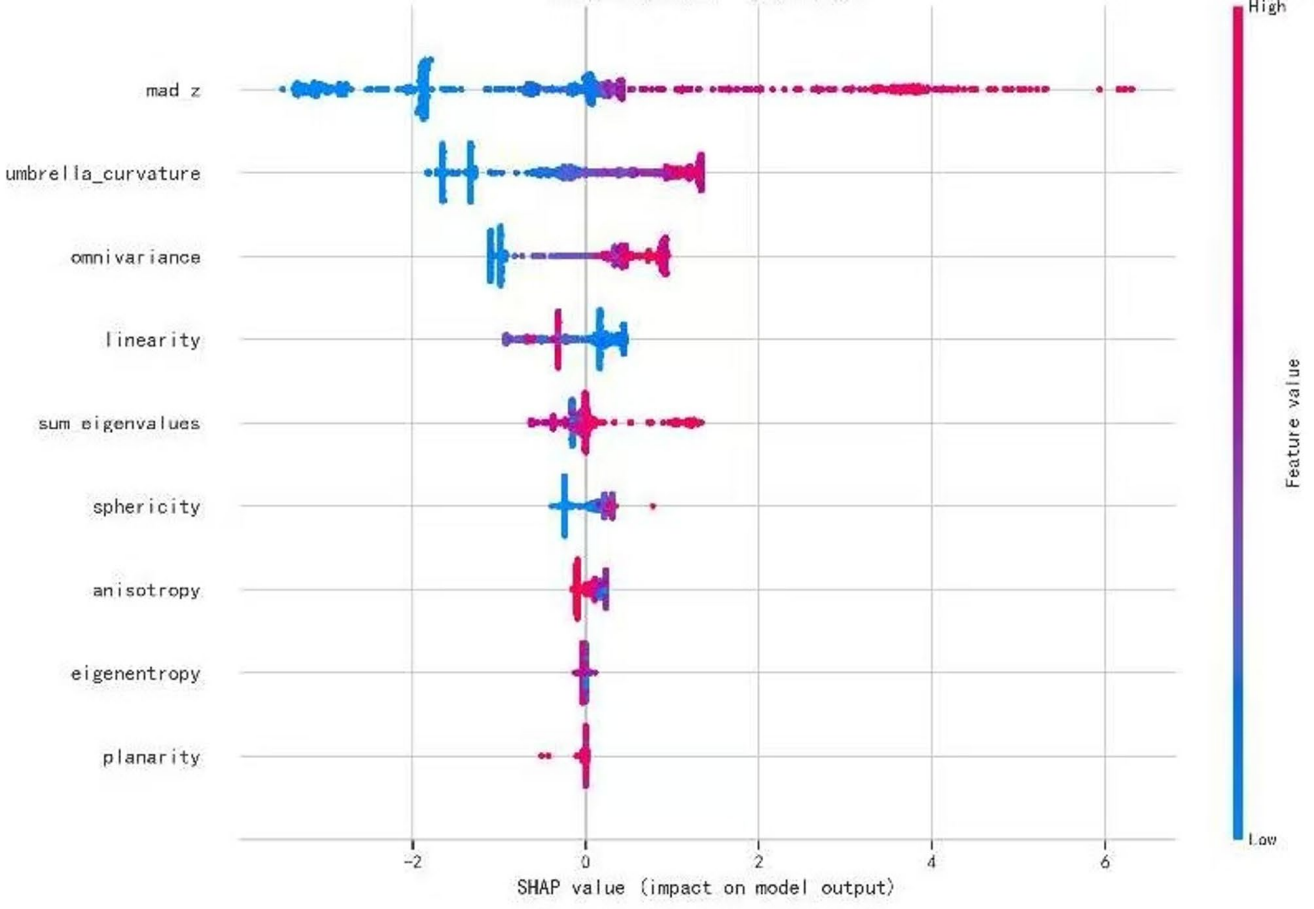
SHAP summary plot for the straight-line weld dataset. Red indicates high feature values, and blue indicates low feature values.

For the straight-line weld dataset, Figs. 21 and 22 present the feature importance ranking and the SHAP summary plot, respectively. The results show that mad_z has the highest relative importance, followed by features such as umbrella curvature and omnivariance. Their SHAP distributions indicate that these features have a stronger influence on the classification output under the present model setting. In contrast, features such as eigenentropy and planarity show relatively smaller SHAP magnitudes, suggesting a weaker contribution to the straight-line weld classification results.

**Fig. 23:**
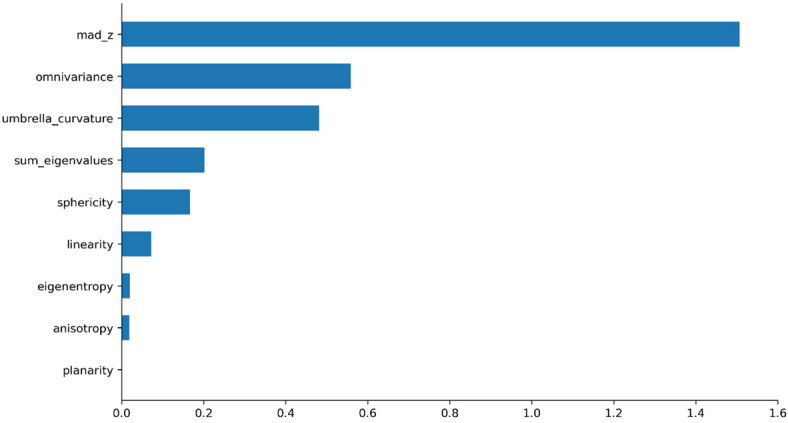
Geometric feature importance ranking for the curved-line weld dataset.

**Fig. 24:**
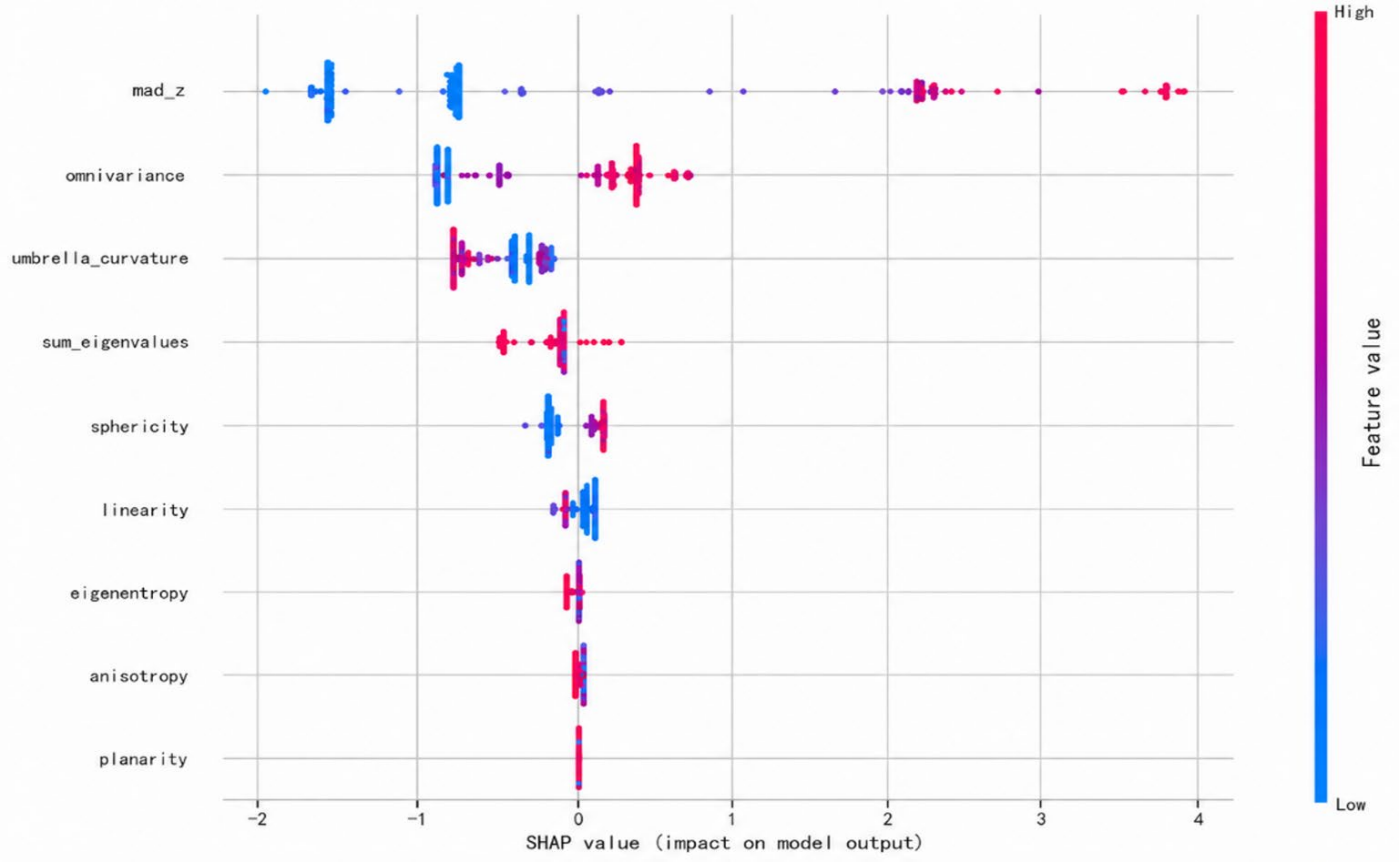
SHAP summary plot for the curved-line weld dataset. Red indicates high feature values, and blue indicates low feature values.

For the curved-line weld dataset, Figs. 23 and 24 show the corresponding feature importance ranking and SHAP summary plot. Similar to the straight-line case, mad_z remains the most influential feature, while omnivariance and umbrella curvature also rank highly. Compared with the straight-line dataset, the relative contributions of anisotropy, eigenentropy, and planarity are lower, and their SHAP values are more concentrated near zero, indicating that their influence on the prediction output is relatively limited under the present setting.

**Fig. 25:**
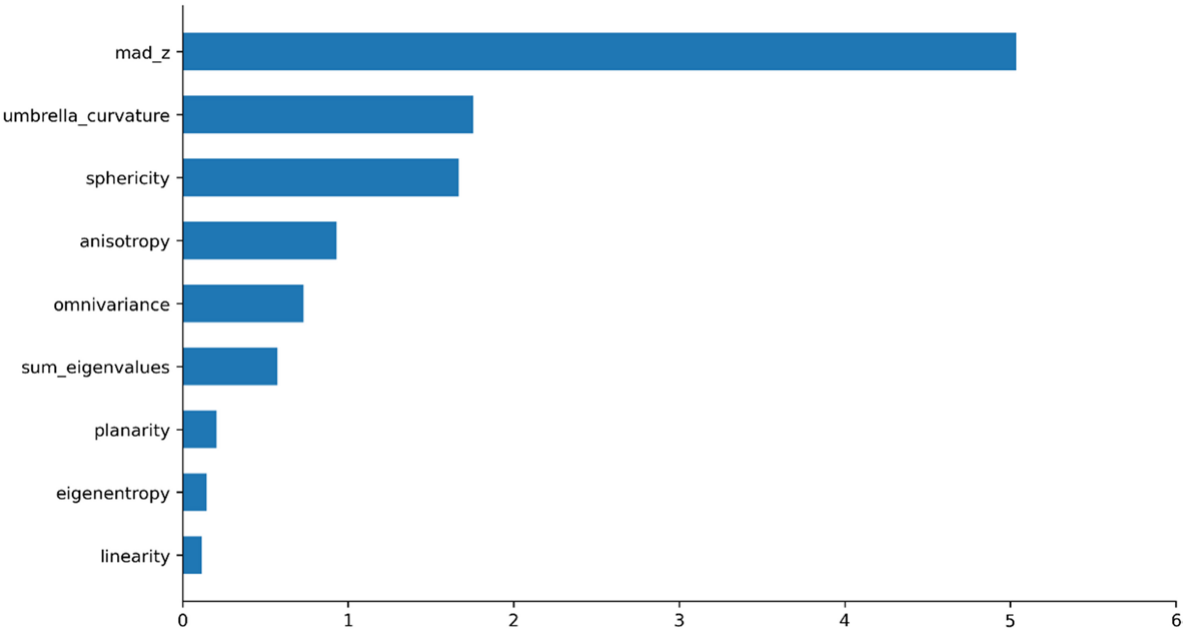
Geometric feature importance ranking for the S-shaped weld dataset.

**Fig. 26:**
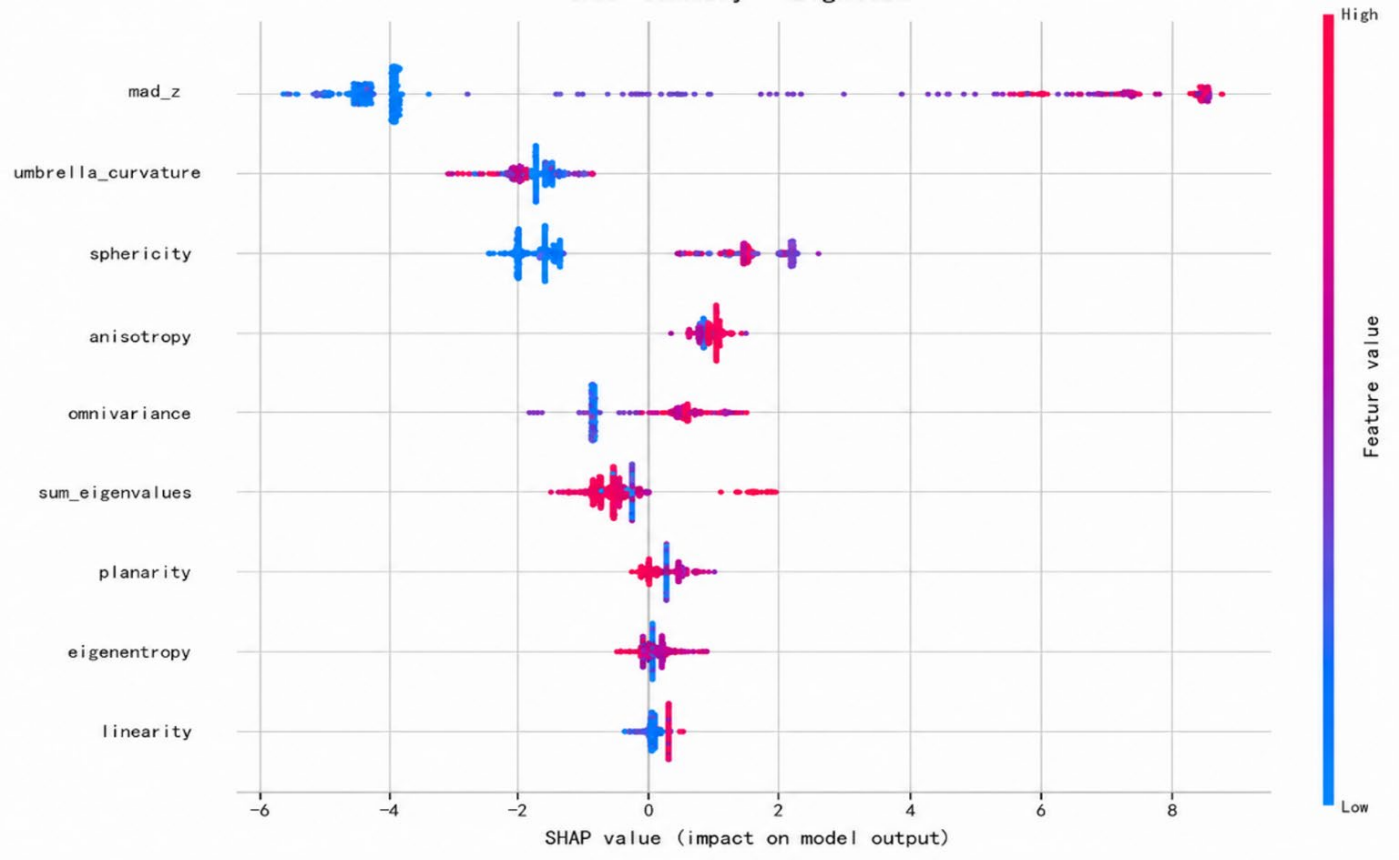
SHAP summary plot for the S-shaped weld dataset. Red indicates high feature values, and blue indicates low feature values.

For the S-shaped weld dataset, Figs. 25 and 26 present the feature importance ranking and the SHAP summary plot. The results again show that mad_z, umbrella curvature, and omnivariance are the most influential features. By contrast, linearity, eigenentropy, and planarity exhibit relatively smaller SHAP magnitudes, indicating that they contribute less to the prediction results of the current model. Across the three morphology-specific datasets, the overall trend remains consistent, namely that height-related fluctuation and local surface-shape descriptors play a more important role in distinguishing weld-region points from base-metal points.

Overall, the feature importance rankings and SHAP summary plots improve the interpretability of the optimized AE-LightGBM model by revealing the relative roles of different geometric features in weld point-cloud classification. The present results mainly provide an interpretation of feature contributions, while conclusions regarding feature removal or model simplification would require further validation.

### Discussion

The experimental results indicate that AE-LightGBM achieves the most favorable overall performance among the compared models on the straight-line, curved-line, and S-shaped test subsets. After the neighborhood radius was fixed at 1.5 mm, LightGBM provided the best baseline performance among the three machine learning models, and further hyperparameter optimization led to additional improvement in the classification results. Compared with ALA-LightGBM and SFOA-LightGBM, AE-LightGBM consistently obtained higher average classification metrics across repeated runs, suggesting that AE is better suited to the hyperparameter optimization of LightGBM for the present weld point-cloud classification task.

The repeated-run results, statistical significance analysis, and convergence curves together provide a more reliable basis for this conclusion than a single-run comparison. The Wilcoxon signed-rank test with Holm correction shows that AE-LightGBM differs significantly from both ALA-LightGBM and SFOA-LightGBM, while no statistically significant difference is observed between ALA-LightGBM and SFOA-LightGBM. In addition, the convergence analysis shows that AE maintains a more favorable optimization trajectory, with faster improvement in the intermediate stage and more stable behavior in the later stage. These findings indicate that AE offers a more effective balance between exploration and exploitation during hyperparameter search.

From the perspective of practical application, the efficiency results further show that AE-LightGBM maintains competitive computational cost while achieving the best overall classification performance. This is particularly relevant for robot-oriented weld recognition and grinding tasks, where both predictive accuracy and computational efficiency are important. The present results therefore suggest that AE-LightGBM is a promising approach for weld point-cloud classification under the studied experimental conditions.

The SHAP-based analysis also provides useful insight into the relative contributions of different geometric features to the classification results, thereby improving the interpretability of the optimized model. At the same time, the present study is based on the current dataset, feature extraction scheme, and acquisition conditions. Further validation on more diverse weld datasets and industrial scenarios would be valuable for extending the applicability of the proposed approach. In addition, more detailed studies on explanation stability and feature ablation may further strengthen the interpretability analysis in future work.

## Conclusions

This study investigated a binary point-wise classification task for weld point clouds and evaluated machine learning models on three representative weld morphologies, namely straight-line, curved-line, and S-shaped welds. The results show that, when the neighborhood radius was set to 1.5 mm, LightGBM achieved the best baseline performance among RF, XGBoost, and LightGBM.

To further improve classification performance, ALA, AE, and SFOA were introduced for LightGBM hyperparameter optimization. Among the three optimized models, AE-LightGBM achieved the best overall performance on the three morphology-specific test subsets. The repeated-run statistical results, significance analysis, and convergence analysis together indicate that AE provides the most favorable overall optimization results among the compared methods for the present task.

In addition, SHAP-based analysis was employed to examine the relative contributions of geometric features to the classification results, which further improves the interpretability of the optimized model. The efficiency evaluation also shows that AE-LightGBM maintains competitive computational cost while providing the best overall classification performance. These results suggest that the proposed method has good potential for weld recognition and robot-assisted grinding applications based on 3D vision.

Although the present results are encouraging, further studies on more diverse industrial environments, larger-scale datasets, and more comprehensive interpretability and feature ablation analyses would be valuable in future work.

## Data Availability

The weld bead 3D point cloud dataset generated in this study can be made available from the corresponding author upon reasonable request.
